# Advance care planning for adolescents with cancer and their parents: study protocol of the BOOST pACP multi-centre randomised controlled trial and process evaluation

**DOI:** 10.1186/s12887-021-02841-7

**Published:** 2021-09-01

**Authors:** Anne van Driessche, Aline De Vleminck, Joni Gilissen, Marijke C. Kars, Jutte van der Werff ten Bosch, Luc Deliens, Joachim Cohen, Kim Beernaert

**Affiliations:** 1 End-of-Life Care Research Group, Vrije Universiteit Brussel (VUB) & Ghent University, Laarbeeklaan 103, 1090 Brussels, Belgium; 2 Department of Family Medicine and Chronic Care, Vrije Universiteit Brussel (VUB), Laarbeeklaan 103, 1090 Brussels, Belgium; 3 Atlantic Fellow for Equity in Brain Health, Global Brain Health Institute (GBHI), University of California San Francisco (UCSF), California, USA; 4 Center of Expertise Palliative Care, Julius Center of Health Sciences and Primary Care, UMC Utrecht, Universiteitsweg 100, Utrecht, CG 3584 the Netherlands; 5 Department of Paediatric Hematology-Oncology, Vrije Universiteit Brussel (VUB), Universitair Ziekenhuis Brussel (UZ Brussel), Laarbeeklaan 101, 1090 Brussels, Belgium; 6 Department of Public Health and Primary Care, Ghent University, 9000 Ghent, Belgium

**Keywords:** Advance care planning, Paediatric palliative care, Paediatric oncology, Multi-Centre randomised controlled trial, Communication, Adolescent, Parent-adolescent communication

## Abstract

**Background:**

Research has highlighted the need for evidence-based interventions to improve paediatric advance care planning (pACP) in adolescents with cancer. Although adolescents express the desire and ability to share their values, beliefs and preferences for treatment, there is a lack of structured multicomponent interventions to improve parent-adolescent communication on different ACP themes including those not limited to end-of-life care. The aim of this study is to evaluate the effectiveness and implementation, context and mechanisms of impact of a novel ACP program in paediatric oncology.

**Methods:**

We will conduct a multi-centre parallel-group randomised controlled superiority trial with embedded mixed-methods process evaluation in Flanders, Belgium. Adolescents aged 10–18 who have cancer, and their parent(s) will be recruited via all four university hospitals in Flanders, Belgium, and support groups. Families will be randomised to receive care as usual or the multicomponent BOOST pACP program, consisting of three conversation sessions between an external facilitator and the adolescent and parent(s).

The primary endpoint is improved parent-adolescent communication from the perspective of the adolescent. Secondary endpoints are adolescents’ and parents’ attitudes, self-efficacy, intention and behaviour regarding talking about ACP themes with each other, parents’ perspective of shared decision making in the last clinical encounter, and the paediatric oncologist’s intention and behaviour regarding talking about ACP themes with the family.

Measurements will be performed at baseline, at 3 months and at 7 months using structured self-reported questionnaires. We will perform a process evaluation in the intervention group, with measurement throughout and post-intervention, using structured diaries filled out by the facilitators, interviews with facilitators, interviews with involved paediatric oncology teams, and audio-recordings of the BOOST pACP conversations.

**Discussion:**

The BOOST pACP program has been developed to stimulate conversations on ACP themes between parent(s) and the adolescents, simultaneously lowering the threshold to discuss similar themes with healthcare professionals, initiating a process of normalization and integration of ACP in standard care. This combined outcome and process evaluation aims to contribute to building the necessary evidence to improve ACP in paediatric oncology.

**Trial registration:**

The study is registered at ISRCTN, ISRCTN33228289. Registration date: January 22, 2021.

**Supplementary Information:**

The online version contains supplementary material available at 10.1186/s12887-021-02841-7.

## Background

### Background and rationale

In Europe, cancer is one of the leading causes of death in children and adolescents [1]. A recent study from the Netherlands [1] found that the overall cancer incidence increased by an average of + 0.6% annually over a 28-year period (from 1990 to 2017). This increase was seen particularly in infants and young adolescents [1, 2]. In Belgium, every year about 340 children (0–14 years) and 180 adolescents (15–19 years) are diagnosed with a malignancy [2]. Although survival rates for childhood cancer have steadily improved over the last few decades from 58% in the mid-1970s to over 80% today [3], disclosure of the diagnosis and subsequent treatment can still have a significant emotional impact on adolescents and parents and poor psychosocial outcomes that may in turn impact the well-being of the entire family [3].

In paediatrics, treatment decisions are routinely made by surrogates, in most cases parents [4]. Therefore, it is important that parents are aware of their child’s preferences for care and their underlying values. The process of advance care planning (ACP) has been promoted as a successful strategy for communication between patients, surrogate decision-makers and healthcare professionals [5]. It is defined as a voluntary process of discussion and review enabling patients to express their views, values and specific treatment choices to inform their future care [6]. Studies in adults demonstrate a range of positive outcomes, including increased congruence between treatment preferences expressed by the patients and their caregivers and increased likelihood that these preferences will be honoured at the end of life [7]. Limited evidence in adolescents with serious illnesses has demonstrated that they have the desire and ability to share their values, beliefs and preferences for treatment at the end of life [8, 9]. In addition, although parents report finding it difficult to engage in ACP with their child, they consider it important, arguing for a sensitive, individualized and gradual approach where hope and quality of life issues are paramount [10].

Although international guidelines and medical societies such as The American Academy of Paediatrics, the Institute of Medicine, and the World Health Organization (WHO) strongly recommend ACP in paediatrics [11], research on specific ACP tools or programs is scarce. The few tools and programs concerning paediatric ACP that do exist [12–18], either lack robust evidence of their effectiveness, or are focused at documentation of end-of-life care preferences; c.q. advance directives, ignoring recent recommendations about ACP being a comprehensive communication process focusing on current and future care [19].

Engaging in ACP can provide an opportunity to address misconceptions about values, preferences and goals regarding current and future care, can improve understanding of the adolescent’s prognosis, can contribute to preparing for future situations [20–22], and can lead to an increased sense of control and security in adolescents [10]. The limited evidence available suggest that families benefit from enhanced communication around ACP and the end of life, both among family members and between the family and the medical team [23]. However, we currently lack robust studies that have evaluated the effectiveness of pACP interventions that do not primarily address end-of-life care preferences.

We developed the BOOST pACP program (Benefits of Obtaining Ownership Systematically Together in paediatric Advance Care Planning) to improve parent-adolescent communication on pACP themes for a heterogeneous population of adolescents (age 10–18) with cancer, by discussing a broad array of ACP themes not limited to end-of-life care and applying a structured format.

### Objectives

We aim to evaluate:

1. The effectiveness of the BOOST pACP program on parent-adolescent communication about ACP themes, self-efficacy towards and attitude on talking about ACP themes with each other.
2. The implementation process of the BOOST pACP program, important contextual factors and mechanisms of action.

In reporting the study protocol, we followed the Standard Protocol Items of the Recommendations for Interventional Trials (SPIRIT) 2013 Checklist [24].

## Methods

### Study design

A multi-centre parallel-group randomised controlled superiority trial [25] (RCT) with embedded mixed-methods process evaluation, comparing the BOOST pACP program to standard care (control group). Adolescent patients and their parent(s) will be randomised with a 1:1 allocation ratio to the intervention arm or the control arm after baseline assessment. The mixed-methods process evaluation is structured according to the Medical Research Council (MRC) Framework by Moore et al. [26].

### Study setting and population

The study will take place in four participating university hospitals in Flanders, Belgium. Families in the intervention group have the choice of having the ACP conversations and visits from the data collector at home, in the hospital or online due to COVID-19 measures. The BOOST pACP program is targeted at adolescents, their parent(s) and involved paediatric oncologists. Inclusion- and exclusion criteria can be found in Table 1. Adolescents should be age 10–18, diagnosed with any type of cancer at any stage and currently receiving (active) oncology treatment. ‘Active treatment’ encompasses adolescents: 1) who are currently receiving treatment; 2) for whom treatment is planned; 3) for whom the last treatment took place maximally 3 months ago.

**Table 1:** Inclusion and exclusion criteria of adolescents, parent(s) and paediatric oncologists

Inclusion criteria (combined by ‘AND’)	Exclusion criteria (combined by ‘OR’)
*Adolescent*	
Aged between 10 and 18 years	Participated in the feasibility test
Diagnosis of cancer ≥3 months prior to inclusion	Intellectual disabilities to an extent that general communication is very difficult, or serious mental health problems to an extent that the extra effort of participating in the study is not justified (estimated by the paediatric oncologist and psychologist)
Aware of, or informed about, cancer diagnosis according to parent(s)	Life expectancy ≤3 months
Receiving active treatment in a paediatric oncology ward	Not receiving active treatment (last treatment was more than 3 months ago)
Fluent Dutch language understanding	
*Parent(s)*	
Aware of, or informed about the diagnosis of their child according to the clinician	Participated in the feasibility test
Fluent Dutch language understanding	Intellectual disabilities to an extent that general communication is very difficult, or serious mental health problems to an extent that the extra effort of participating in the study is not justified (estimated by the paediatric oncologist and psychologist)
*Paediatric oncologist*	
Medically involved in the treatment of the adolescent, indicated by the family to be the oncologist with whom the family has most contact about treatment	
Fluent Dutch language understanding	

**Fig. 1:**
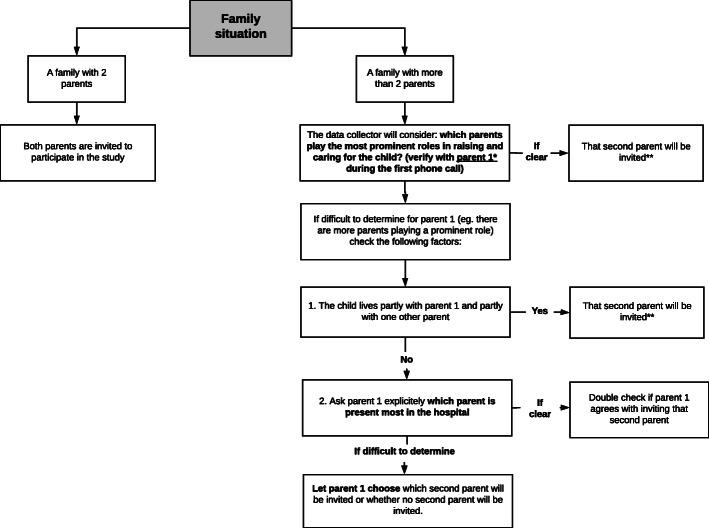
Flow diagram to identify and invite parents for the BOOST study. *parent 1 – parent with whom the data collector had contact about the study. **except when parent 1 does not want to participate together with that second parent

A maximum of two of the most involved parents will be invited to take part; the term “parent/s” includes legal guardians and stepparents. To determine which parents are to be invited into the study, data collectors will follow a flow diagram (Fig. 1). In case there is only one parent, he/she will automatically be included. If there are more than two parents in the adolescent’s life (e.g. due to blended families), the two parents with the most prominent role in raising and caring for the child will be invited to take part in the study. If the latter is difficult to determine, the most prominent parent will decide which second parent will be invited to participate in the study. The most prominent parent is the one that is usually present and accompanies the adolescent to the hospital and during oncology care and treatment, and also identifies himself or herself as the most prominent parent.

### Intervention and control arm

#### Intervention: the BOOST pACP program

The “BOOST pACP” (**B**enefits of **O**btaining **O**wnership **S**ystematically **T**ogether in **paediatric A**dvance **C**are **P**lanning) program will be provided alongside any standard care in the intervention group. The main goal of the program is to improve communication about ACP themes between adolescents with cancer and their parents.

The program was developed by following a comprehensive iterative approach, informed by the supplementary guidance for the development of complex interventions by Bleijenberg et al. [27], in addition to the MRC Framework [28]. Using input from interviews with healthcare professionals and a review of the grey literature and the academic literature via PubMed, we developed a conceptual framework outlining the pathways through which the intervention would change desired outcomes. We then identified a pACP program in the Netherlands, called IMPACT (Implementing Paediatric Advance Care Planning Toolkit) [16], that matched the direction of the BOOST pACP program as defined by the conceptual framework. The IMPACT preparation booklets, the summary sheet and conversation structure were integrated into and adapted to the BOOST pACP program, in close collaboration with the developers of IMPACT (MK, JF). All components were adapted in language and content to match the Flemish context and the specific goals and population of the program. Conversation cards were developed to structure the conversations, integrating a method that has been shown to facilitate open communication [29–31]. All materials were tested for acceptability and feasibility with adolescents and young adults (AYAs) with cancer (*n* = 4), parents (*n* = 6) and healthcare professionals (*n* = 9), and adapted accordingly. The first BOOST pACP conversation session and transfer of information to the paediatric oncologist was tested with three families.

The program is described in Table 2, according to the TIDieR (template for intervention description and replication) Checklist [32].

**Table 2:** Summary of BOOST pACP program according to the Template intervention description and replication (TiDieR) checklist

TiDieR number	TiDieR item	BOOST pACP program
1.	BRIEF NAME (name or a phrase that describes the intervention)	BOOST pACP (Benefits of Obtaining Ownership Systematically Together in paediatric advance care planning) program
2.	WHY (any rationale, theory, or goal of the elements essential to the intervention)	A more positive attitude and improved self-efficacy towards communicating about ACP themes between adolescents and their parent(s) may result in an intention to talk more about ACP themes. This in turn may lead to improved parent-adolescent communication.
3.	WHAT Materials (any physical or informational materials used in the intervention, including those provided to participants or used in intervention delivery or in training of intervention providers)	The BOOST pACP program includes 8 supporting materials: 1. Training program and accompanying PowerPoint presentations used for the 2.5-day training of the ACP facilitators 2. A manual for trained ACP facilitators who will perform the ACP conversations with adolescents and parent(s), including a description of steps to follow during and between the structured ACP conversation sessions. 3. Preparation booklets for both the adolescent and parent(s), including information about the program, why ACP is relevant and questions to trigger the thinking process about ACP themes. 4. A video in which two families talk about their personal situation and experienced effects of the ACP program. 5. Conversation cards to structure the ACP conversation sessions with the adolescent covering the following ACP themes: 1) Who am I?; 2) How do I experience my illness? 3) Talking with others; 4) Help and comfort; 5) Worries and fears; 6) What care do you want?; 7) Expectations for the future; 8) About dying. Adolescents are asked to divide the themes into two categories: “I would like to talk about this theme with my parent(s)” and “at the moment I do not feel the need to talk about this theme with my parent(s)” 6. Conversation cards to structure the ACP conversation session with parent(s) covering the following ACP themes: 1) Talking with your child; 2) Parenthood; 3) Help and comfort; 4) Worries and fears; 5) Care and treatment; 6) Expectations for the future; 7) About dying 7. A summary sheet that will be filled out by the parent(s) and adolescent together in session 3 to stimulate conversation about ACP themes. 8. Conversation cards that can be used as a game of quartet at home. Families will receive these cards at the end of session 3.
4.	WHAT Procedures (each of the procedures, activities, and/or processes used in the intervention, including any enabling or support activities)	The BOOST program entails 10 program components that can be carried out via 17 activities: **As part of “Facilitator training (including manual)” component (1)** Activity 1: Selection of two external facilitators Activity 2: Preparation of facilitators for the training Activity 3: Two-and-a-half-day training program for facilitators Activity 4: Intervision sessions (discussing challenges in case studies) approximately every 4 months for facilitators (2–5 h) **As part of “Preparation booklets” component (2)** Activity 5: Data collectors give preparation booklets to the families that are assigned to the intervention group Activity 6: Adolescent and parent(s) read their preparation booklet before conversation session 1 takes place **As part of “Video” component (3)** Activity 7: The facilitator introduces the videos that will be shown in conversation session 1. Activity 8: Adolescent and parent(s) watch the video during conversation session 1. Activity 9: The facilitator asks whether the adolescent and parent(s) recognize any aspect from the video. **As part of “Conversation session 1” (4)** Activity 10: The facilitator guides the conversation session and introduces the conversation cards to the adolescent and parent(s): **As part of “Conversation session 2a” (5)** Activity 11: The facilitator guides the conversation session with the adolescent alone and uses conversation cards to discuss ACP themes. **As part of “Conversation session 2b” (6)** Activity 12: The facilitator guides the conversation session with the parent(s) alone and uses conversation cards to discuss ACP themes. **As part of “Conversation session 3” (7)** Activity 13: The facilitator guides the conversation session with the adolescent and parent(s) and allows them to discuss ACP themes. **As part of “Summary sheet” (8)** Activity 14: The facilitator introduces and explains the summary sheet. The facilitator asks whether the family would like the information they write down to be shared with the paediatric oncologist. **As part of “Conversation cards that can be used as a game of quartet at home” (9)** Activity 15: The facilitator explains the purpose of the quartet game and that the cards can facilitate communicating on ACP themes together at home in a playful way. **As part of “Transfer of information to a treating paediatric oncologist” (10)** Activity 16: In case the family gave permission, the facilitator makes an appointment with the paediatric oncologist that the family indicated. Activity 17: The facilitator gives a summary of the conversations with the family to the paediatric oncologist and asks whether the paediatric oncologist can add the summary sheet to the electronic dossier of the patient.
5.	WHO PROVIDED (intervention provider, their expertise, background and any specific training given)	External facilitators are hired by the research team to perform the BOOST pACP program, more specifically to facilitate the structured ACP conversations with the families. Facilitators (psychologists) will receive a 2.5 day training and ongoing 4-monthly intervision to respond to different scenarios and situations (such as strategies when there is little response, dealing with resistance, and emotional feelings of the participants).
6.	HOW (modes of delivery)	All ACP conversation sessions are provided face-to-face (at home or at the hospital) or online by video call, depending on the family’s preference. The transfer of information with the paediatric oncologist/ medical team will take place by (video) call or in real life.
7.	WHERE (the type(s) of location(s) where the intervention occurred, including any necessary infrastructure or relevant features)	The conversation sessions can take place either at the home address of the family, in the hospital or online by video call (because of the COVID-19 measures).
8.	WHEN and HOW MUCH (the number of times the intervention was delivered and over what period of time including the number of sessions, their schedule, and their duration, intensity or dose)	The intervention period per family is three months (from receiving the preparation booklets to the transfer of information with the paediatric oncologist). Each conversation session will take approximately 60 min. There will be at least one week in between the conversation sessions, due to the importance of time to reflect on ACP themes and potentially discuss certain ACP themes at home if participants want to. The intervision sessions for the facilitators will take maximally five hours and will be planned every 4 months.
9.	TAILORING (if the intervention was planned to be personalized, titrated or adapted, then describe what, why, when, and how)	Within the conversation sessions, participants have freedom and flexibility in choosing certain ACP themes to discuss and the facilitator will clarify that participants are not obligated to talk about all ACP themes. The conversation sessions are structured in the sense that per topic, there is a key question and two follow-up questions that will be asked always. Apart from that, facilitators can ask other questions or decide to zoom in on the participant’s story whenever they want. Families can choose the mode of delivery of the conversation sessions (face-to-face at home or in the hospital or online by video call). Furthermore, the facilitator will ask paediatric oncologists how they would like to organize the transfer of information (if family permitted sharing a summary of the conversations with healthcare professionals).
10	FIDELITY - HOW WELL (planned)	As part of the process evaluation, we will record audio recordings of all structured ACP sessions 1, 2a, 2b and 3 from eight dyads in total (after consent) to evaluate fidelity. We will use a fidelity checklist that is based on the steps described in the manual of the facilitators.

The BOOST pACP program consists of ten key components:

1. facilitator training, including manual
2. preparation booklets, sent to the home address of the parent(s) and adolescent
3. a video of two families talking about the experiences with the program, which will be shown during the first conversation session
4. facilitated conversation session 1 (involvement of both the adolescent and parent(s)), aimed at informing the family about ACP and the BOOST program, and stimulating a positive attitude and self-efficacy towards talking about ACP themes with each other. The session is partly structured by using conversation cards.
5. facilitated conversation session 2a (involvement of the adolescent), aimed at exploring the adolescent’s views on ACP themes and what themes he or she would like to talk about with his or her parent(s) and how to talk about them. Session 2a is structured by using conversation cards.
6. facilitated conversation session 2b (involvement of the parent(s)), aimed at exploring the parents’ views on ACP themes and what themes they would like to talk about with their child. Session 2b is structured by using conversation cards.
7. facilitated conversation session 3 (involvement of both the adolescent and parent(s)), aimed at revisiting ACP themes by filling out a summary sheet and leaving room for further discussion of ACP themes if preferred by the family.
8. a summary sheet that will be filled out together by the adolescent and their parent(s), during conversation session 3.
9. conversation cards that can be used as a game of quartet at home.
10. transfer of information from the ACP facilitator to the paediatric oncologist involved in the care of the adolescent.

**Table:** 

Box 1 Themes discussed during structured ACP conversations session 1 to 3	
- For adolescents: 1) identity; 2) experiences with the disease and treatment; 3) talking with others; 4) help and comfort; 5) worries and fears; 6) preferences for care and treatment; 7) expectations for the future; 8) dying. - For parent(s): 1) talking with your child; 2) parenthood; 3) help and comfort; 4) worries and fears; 5) care and treatment; 6) expectations for the future; 7) dying. - Adolescents and parents can choose not to discuss certain themes they may prefer not to talk about.	

**Fig. 2:**
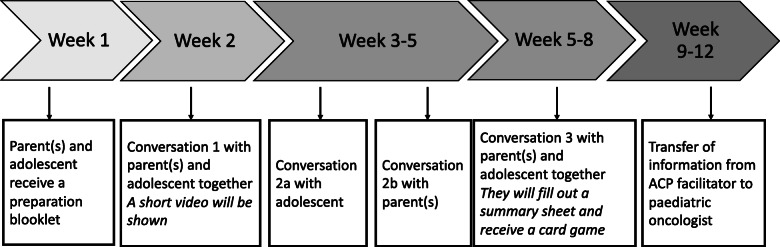
Timeline of the BOOST pACP program with adolescents that have cancer and their parents

Figure 2 shows the timeline of the BOOST pACP program, graphically displaying the different components. Some components coincide in time. There will be approximately one to 2 weeks in between the three conversation sessions, so that adolescents and parents have time to reflect on the themes discussed. The transfer of information from the facilitator to the paediatric oncologist involved in the care of the adolescent will take place within 1 month after conversation session 3.

External facilitators will be hired by the research team to deliver the BOOST pACP program. These facilitators will receive a 2.5-day training, partly provided by a communication expert from an external firm (Wilde Kastanje Training and Education, the Netherlands) in performing ACP conversations with the adolescent and parent(s) separately and with the family members together. The facilitators meet the following criteria: Master’s degree in Psychology, advanced communication skills, and experience in working with adolescents.

#### Control

The control group will not receive the BOOST pACP program. Adolescents in the hospitals are seen by different oncologists and nurses. Families that are recruited via four participating university hospitals will have access to contact with psychologists whenever they request it. Paediatric ACP is not implemented as standard care within these hospitals. The conversation cards that can be used as a game of quartet at home are offered to the families in the control group after study completion.

#### Criteria for discontinuing or modifying allocated interventions

Families can withdraw from the study at any moment. Where a participant experiences psychological distress the facilitator, being an experienced psychologist by background, will discuss their feelings and ask if they want to continue. In addition, if a participant displays signs of psychological distress, the ACP facilitator will discuss and facilitate contact with the psychologist from the involved paediatric oncology wards. Where patients are recruited through support organizations, the facilitator will ask the family which psychologist from their facility they can contact.

A bereavement protocol will be in place for when a patient or parent dies during the study period. When the data collector is notified about the death of a participant, he/she will then notify the trial manager and ACP facilitator. The bereaved participant will receive a phone call from the data collector outlining condolences and where they can access support if required. Bereaved participants will not be asked or expected to complete follow-up study questionnaires.

#### Strategies to improve adherence to the intervention protocols

The research team also developed an implementation protocol outlining all intervention components and associated procedures, in collaboration with the facilitators that they use throughout the delivery of the BOOST pACP program. Next to the 2.5-day training, the ACP facilitators will participate in 4-monthly (or more, depending on need) intervisions/comeback sessions (‘coaching on the job’) with a researcher (AVD) and a specialist trainer (in communication) from the research group. Intervisions are aimed at providing facilitators with additional knowledge and skills required to successfully implement the program, based on a discussion of the challenges they experience, and implementation approaches and strategies to deal with these challenges.

#### Relevant concomitant care and interventions that are permitted or prohibited during the trial

There are no restrictions regarding concomitant care or interventions during the trial.

### Outcomes

Outcomes are measured at baseline (T0), 3 months after baseline (T1), and at 7 months (T2) in three groups: adolescents, parents and paediatric oncologists. The outcome measures used for the primary and secondary endpoints are shown in Table 3.

**Table 3:** Outcome measures of the primary and secondary endpoints of the BOOST pACP program

Outcomes	Measurement instrument	No. of items	Unit of analysis	Timepoint		
				T0	T1	T2
**Primary endpoint**						
1. Quality of Parent-Adolescent Communication	Parent-Adolescent Communication Scale (PACS) [32]	20 items	Adolescent		x	
**Secondary endpoints**						
2. Quality of Parent-Adolescent Communication	PACS	20 items	Adolescent	x		x
3. Attitude on talking with the other (parent(s)/ their child) about what the adolescent finds important regarding his/her care and treatment	Created by the research team^a^	8 items	Adolescent	x	x	x
		7 items	Parent(s)	x	x	x
4. Self-efficacy towards talking with the other (parent(s)/ their child) about different ACP themes	Created by the research team^a^	9 items	Adolescent	x	x	x
4a. if the child initiates the conversation	Created by the research team^a^	9 items	Parent(s)	x	x	x
4b. to initiate the conversation his/herself	Created by the research team^a^	9 items	Parent(s)	x	x	x
5. Self-efficacy towards talking with their child and letting their child talk in different situations	Created by the research team^a^	6 items	Parent(s)	x	x	x
6. Behaviour: talking with the other (parent(s)/ their child) about different ACP themes	Created by the research team^a^	9 items	Adolescent	x	x	x
		9 items	Parent(s)	x	x	x
7. Intention to talk with the other (parent(s)/ their child) parent(s) about different ACP themes	Created by the research team^a^	9 items	Adolescent	x	x	x
		9 items	Parent(s)	x	x	x
8. Behaviour: talking with paediatric oncologist about different ACP themes	Created by the research team^a^	9 items	Adolescent	x	x	x
9. Intention to talk with paediatric oncologist about different ACP themes	Created by the research team^a^	9 items	Adolescent	x	x	x
10. Anxiety	PROMIS Anxiety 8a Short version [33]^b^	8 items	Adolescent	x	x	x
	PROMIS 7a Short version [34]^b^	7 items	Parent(s)	x	x	x
11. Level of shared decision making	CollaboRATE Scale [35, 36]^c^	3 items	Parent(s)	x	x	x
12. Satisfaction items for the intervention group	Created by the research team	14 items	Adolescent and parent(s)		x	x
13. Behaviour & intention to discuss ACP with the family	Created by the research team	3 items	Oncologist	x	x	x
14. Quality of Life	EQ-5D-Y [37]	6 items	Adolescent	x	x	x
**Background characteristics**						
15. Demographic and background information	Created by the research team	3 items	Adolescent	x		
		14 items	Parent(s)	x		
16. Disease & treatment information about adolescent	Created by the research team	7–10 items	Oncologist	x	x	x

^a^The following procedure was followed: 1) The Theory of Planned Behaviour instructed ways items were formulated regarding the four key constructs operationalizing ACP as a health behaviour: attitude, self-efficacy, intention and actual behaviour; 2) the prototype instrument was linguistically improved and made age appropriate by a literacy expert agency; 3) cognitive interviews with adolescents who were diagnosed with cancer (*n =* 4) and parents (*n =* 6) were then performed; 4) feedback using thematic analysis and discussions with the researchers informed refinements. Response categories: ‘Strongly Disagree’, ‘Disagree’, ‘Neither Agree Nor Disagree’, ‘Agree’ or ‘Strongly Agree’

^b^Response categories: ‘Never’, ‘Rarely’, ‘Sometimes’, ‘Often’, or ‘Always’.

^c^Response categories: 10 – point Likert scale, ranging from ‘No effort was made’ (=0) to ‘Every effort was made’ (=10)

The full questionnaires used at T0 in these three groups are provided in Additional file 1.

#### Primary endpoint

The primary endpoint is dyadic ACP communication (between adolescent and parent) at 3 months (T1), using the Parent-Adolescent Communication Scale (PACS) [38], a validated questionnaire to measure the adolescent’s perception of their communication, as applied to pACP themes with (both of) the parent(s). The Parent-Adolescent Communication Scale [38] entails two subscales: 1) openness in communication subscale; 2) problems in communication subscale, including 20 items, that can be rated from 1 to 5 and produce factor-derived scores (range: 10–50). Items are summed for each scale, so that higher scores indicate more openness and more problems. The scores for items on the Problems subscale are reversed in value. The score ranges from 20 to 100 on the full scale. Both subscales have demonstrated strong internal consistency (alpha = 0.87–0.78) and test-retest reliability (r = 0.78–0.77) [37]. The questionnaire was translated to Dutch following a forward-backward translation procedure, in collaboration with an independent translation agency. The adolescent will fill out the PACS about both parents, if applicable.

#### Secondary endpoints

One of the secondary endpoints is dyadic communication (between adolescent and parent(s) at 7 months (T2). The other secondary endpoints will be measured at baseline (T0), T1 and T2 and include:

- adolescent’s/parents’ attitudes to talking about ACP themes with his/her parent(s)/child (self-developed items)
- adolescent’s/parents’ self-efficacy towards talking about ACP themes with his/her parent(s)/child (self-developed items)
- parents’ self-efficacy towards talking with their child and letting their child talk in different situations (self-developed items)
- adolescent’s/parents’ behaviour of talking about ACP themes with his/her parent(s)/child (self-developed items)
- adolescent’s/parents’ intention to talk about ACP themes with his/her parent(s)/child (self-developed items)
- adolescent’s behaviour of talking about ACP themes with his/her paediatric oncologist (self-developed items)
- adolescent’s intention to talk about ACP themes with his/her paediatric oncologist (self-developed items)
- adolescent’s quality of life (EQ-5D-Y [34])
- parents’ perception of shared decision making in the last clinical encounter (CollaboRATE Scale [33, 36])
- adolescent’s/parents’ level of anxiety in the past week (PROMIS Anxiety [34, 39])
- paediatric oncologists’ behaviour of talking about ACP themes with the family (self-developed items)
- paediatric oncologists’ intention to talk about ACP themes with the family (self-developed items).

#### Participant characteristics

We will collect demographic and relevant background information from adolescents (i.e. gender, data and country of birth) and from parents (i.e. gender, date of birth, highest education). From paediatric oncologists, we will gather relevant clinical information about the adolescent (i.e. main diagnosis, recurrence, metastasis and type and line of treatment).

#### Process evaluation

Via a mixed-methods process evaluation in the intervention group, we will assess:

- implementation: the structures, processes and resources through which delivery is achieved, and the quantity and quality of what is delivered [26]. Outcomes involve: a description of how the program activities were implemented, resources and dose, reach, fidelity, adaptations and quality.
- mechanisms of impact: the intermediate mechanisms through which program activities produce intended (or unintended) effects, and how activities, and participants’ interactions with them, trigger change [26]. Outcomes involved are responses and interactions with the program, mediators and unanticipated pathways or consequences.
- context: factors external to the program which may influence its implementation, or whether its mechanisms of impact act as intended, and how external factors influence the delivery and functioning of the program [26]. This involves contextual moderators and intention for maintenance.

Additional file 2 provides an overview of all process indicators that are evaluated in the process evaluation, indicating the methods applied and at what time point.

### Participant timeline

**Fig. 3:**
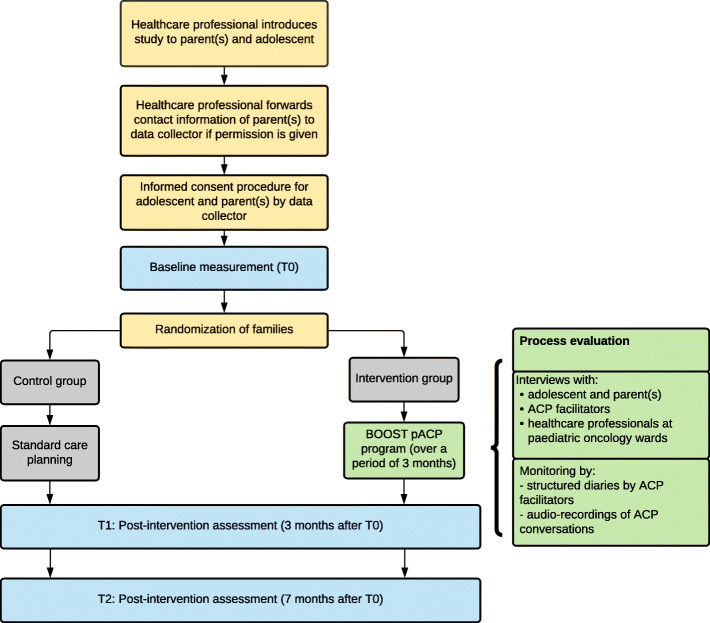
Flow diagram of adolescent and parent recruitment, randomization and measurements

The full trial participation of participants takes up to 7 months (from baseline to filling out the last questionnaire). The flow diagram of measurements is provided in Fig. 3 and the intervention participant timeline is provided in Fig. 2.

### Sample size

We consider demonstration of an intervention effect on the primary endpoint as a success. The primary endpoint is the score for the Parent-Adolescent Communication Scale (PACS) [38] at 3 months (T1).

To detect a minimal relevant difference between intervention and control group at T1 of 7 with a pooled standard deviation in both groups of 11.4, with alpha at 0.05 and 80% power (1-β = 0.80), a sample of *n* = 43 is needed in each arm. Based on the experiences with the FACE intervention [40], we anticipate a 90% retention rate, similar to the FACE intervention, resulting in an estimation of 96 families in total to be enrolled.

Two other studies found a smaller difference in PACS scores between the intervention and control group (e.g. 2.57 [41] and 2.21 [42]). However, both of these intervention studies entailed minimal in-person contact and sending prompts to the parents by mobile phone. The components of the BOOST pACP program entail intensive in-person contact moments, specifically targeted at behavioural constructs to improve parent-adolescent communication on ACP themes. Therefore, we expect to see a larger effect on the PACS scale, compared with the interventions described above.

### Recruitment procedures

The recruitment period will be 2 years (2021–2023). We will recruit adolescents via two different streams:

1. Patients will be recruited in all four participating university hospitals in Flanders, Belgium, via the paediatric oncology wards. Each of the hospital wards will assign one staff member as the main point of contact for the study. The contact person asks each paediatric oncologist to assess which patients would meet the inclusion criteria. The paediatric oncologist or psychologist will ask the parents of these patients whether they would be willing to participate in the study by giving a short explanation and asking whether they agree for a data collector to call them to provide more information about the study. The data collector will then contact one of the parents by phone. If the parent agrees to participate, the data collector will make an appointment with the parent(s) (depending on which parents are considered eligible, see Fig. 1) and the adolescent. During the appointment, the data collector will explain that both parents are encouraged to join the conversation sessions, but that it is not obligatory for both parents to join all conversation sessions of the program to be able to enrol in the study.
2. We will recruit adolescents with cancer and their parent(s) through support organizations that meet the following criteria: 1) they are non-profit; 2) they are based in Flanders or Brussels; 3) adolescents with cancer or their parents are one of their target groups. Organizations are contacted by the researcher (AVD) and are asked to post an open call using a flyer developed by the research team via their newsletters, website and other social media or share this in other ways that are part of their regular channels of communication with their members and network. Adolescents and/or parents can contact the data collectors (via phone or e-mail) when they are interested in participation in the study or when they have additional questions. Subsequent recruitment procedures are similar to the first recruitment stream.

### Allocation

Randomization will be performed after at least one of the parents has completed the baseline measurement. Block randomization with a 1:1 allocation ratio to either the intervention arm or the control arm will be performed, using a concealed envelope method. To ensure equal allocation of subgroups, we will stratify the allocation by age (10–14 years old vs 15–18 years old). We have opted for these age strata, because the cognitive development of adolescents between 10 and 14 years differs from those between 15 and 18 years [43, 44] which might influence the way they are able to talk about their care and treatment.Allocation concealment will be ensured, as the randomization service will not release the randomization code until the adolescent has been recruited into the trial, which takes place after all baseline measurements have been completed. After randomization, the data collector will then inform the families of their allocation to one of the study arms.

#### Masking

Researchers will be single blinded as to which arm families belong to including data analyses. Participating families and paediatric oncologists allocated to the intervention group, and ACP facilitators, cannot be blinded, as they will actively participate in some part of the program. Participants in the control group will subsequently be informed about their group allocation. Research staff responsible for data analyses will not receive any information about a family’s group allocation.

### Data collection procedures

Quantitative data is collected at baseline (T0), after 3 months (T1), and after 7 months (T2) (see Fig. 3), using self-reported questionnaires that are pseudonymized using individual identifier codes attributed by the data collector. During a home visit, the data collector will gather written consent from both parents and written assent from the adolescent, immediately after which the baseline questionnaire is provided to the participant (paper-pencil or online using ‘LimeSurvey’, depending on the choice of the participant). The data collector will fill out the questionnaire together with the adolescent in an interview format. This will be done in a separate room from the parent(s), to avoid that the adolescent is being influenced by their presence. Parent(s) can either choose to fill out their questionnaire independently at the same time or later. The paediatric oncologist mentioned by the family as the one with whom they have most contact about care and treatment will receive a baseline questionnaire about the adolescent via an e-mail link. The data collector will send out one reminder to the parents and paediatric oncologist after 1 week. Data collection procedures are repeated at T1. At T2, questionnaires are sent to parents and adolescents, either online or via postal mail. To reduce loss to follow-up, improve retention, and encourage adolescents to fill out the questionnaires, retention gifts will be provided after completion of each data collection, at T0, T1 and T2.

Data collection procedures for the process evaluation (Additional file 2), include:

1. structured diary filled out by facilitators: the facilitators keep track of all activities they perform regarding the BOOST pACP program by filling out a structured diary, including multiple choice and open-ended questions, after each BOOST pACP conversation session. The diary will be completed in a Word template and will be stored in a secure folder.
2. semi-structured individual interviews with facilitators; both facilitators will be interviewed for 60 min by a researcher at 6 months, 12 months, 18 months and after the end of the data collection period at 24 months.
3. semi-structured individual interviews with a variety of involved paediatric oncology teams after 12 months and after study completion, at 24 months.
4. each BOOST pACP conversation is recorded by audio (after informed consent of participants).

All interviews are structured according to a pre-specified topic list and audio-taped for analysis purposes.

### Data management

All questionnaires will contain a pseudonymizing code consisting of a unique number that is linked to the participant’s name***.*** Data collectors will keep a record of these codes in a secured folder that is not accessible to the researchers. The data collector is responsible for noting the correct unique number of the paper version of the questionnaires at T0, T1 and T2 or for forwarding the code in the email to participants. For the questionnaires of the paediatric oncologists, the data collector will communicate the name of the adolescent with the corresponding code. The paediatric oncologist will be asked to note down the adolescent’s code in the online questionnaire. Data collectors will keep an excel file to indicate to what codes they have sent the questionnaires and share this with researchers. Using this file, the researchers will keep track of responses and will use the unique code to communicate with the data collector to whom a reminder may need to be sent out. Data from paper-and-pencil questionnaires are entered as soon as possible after receipt. Online questionnaires will be automatically saved in a secure open-source web-based survey application (LimeSurvey). Written informed consent files and paper questionnaires are stored in a lockable filing cabinet in a room with restricted access on campus. The file with the participants’ names, address and other identifiable information will be stored in one file. This file will be restricted to the data collectors and ACP facilitators only.

#### Confidentiality

The collected and transferred data will be pseudonymized to ensure that participants’ privacy and personal information are protected. In transcriptions of the interviews, all information that can contribute to tracing and identifying the participants will be removed or replaced. We will use sufficient safety measures to protect the data, including a virtual server firewall, back-up systems and sufficient access controls (i.e., ID and ultrahigh password regulator and frequent password changes).

#### Dissemination

The results of this study will be submitted for publication in peer-reviewed journals and will be presented to the paediatric wards of the participating hospitals and at national and international research and professional conferences. Furthermore, we will disseminate via the website, social media and newsletter of the research group (endoflifecare.be).

### Data analysis

The intervention and control group will be described using descriptive summary statistics (mean, standard deviation, count percentage). We will test for significant differences in the primary and secondary endpoints, between the intervention and control group at baseline and at T1 (3 months from T0), and T2 (7 months from T0) while adjusting for baselines scores.

#### Primary hypothesis testing

The hypotheses related to the primary endpoint, parent-adolescent communication (PACS), will be analysed using a mixed model with the T1 measurement value for parent-adolescent communication as outcome variable and randomization group and baseline measure of parent-adolescent communication as predictor variables. Random effects that we will include in our mixed model are hospital and line of treatment. We will perform analyses on both intention-to-treat and per-protocol principles, with the intention-to-treat as the primary approach. In the intention-to-treat analysis, all participants who were enrolled and randomised will be accounted for in the main analysis, regardless of whether they completed the BOOST pACP program or not. The linear mixed models we intend to apply handle missing data through maximum likelihood estimation (i.e. assuming data is missing at random), so no missing data imputation should be applied. In case we do decide to apply multiple imputation, predictors for the imputation model will include the baseline measurement, randomization group, age and other possibly confounding variables (e.g. prognosis). In the per-protocol analysis, we will focus on families of which at least one parent was involved in all BOOST pACP conversations. During this analysis, we regard the family as a cluster because it is possible that during some conversation sessions both parents are present, while during others only one parent is present. Therefore, we will add the presence of parents during the conversation sessions as time-varying covariates.

#### Secondary hypotheses testing

All secondary endpoints will be evaluated by testing the BOOST pACP program against the control group for each participant population separately (adolescents, parents and paediatric oncologists) with random effects being hospital and line of treatment.

For each of the primary and secondary endpoints, subgroup analyses will be performed using formal interaction tests to explore the extent to which the outcomes of the trial differ by gender, working situation and family situation of the parent(s) and gender and prognosis of the adolescent. Interaction terms between gender, working situation and family situation of the parent(s) and gender and prognosis of the adolescent on the one hand and the trial arms on the other hand will be added to the analysis models.

By including the baseline measurement as a predictor variable pre-existing differences will be controlled, enhancing the sensitivity of the analyses. Alpha is set at 0.05. To interpret the magnitude of the effects for the different outcomes, we will estimate effect sizes (Cohen’s d). Analyses will be conducted in IBM SPSS and R.

#### Process evaluation

Process evaluation of the implementation of the program will be analysed following the domains of Moore et al.’s MRC framework for process evaluations of complex interventions [26]: implementation, causal mechanisms and context, guided by pre-defined process indicators outlined in Additional file 2. Quantitative data collected via structured diaries (e.g. duration of an ACP conversation session) will be summarized by calculating frequencies and descriptive statistics (mean, median, standard deviation, range) using SPSS. All qualitative transcript data from interviews and audiotaped ACP conversations will be analysed by using thematic content analysis (i.e. deductive coding guided by the pre-defined process indicators, e.g. number of activities delivered as intended) and inductive coding into themes [45, 46]. The inductive approach will enable recognition of unexpected emergent themes. Qualitative data analysis will be managed in NVIVO. One researcher will read the transcripts carefully several times to have a sense of the data, with a 10% random sample of transcripts double coded by another researcher.

### Data monitoring

The main researcher (AVD) and data collectors will continuously monitor trial response using MS Excel sheets. An independent trial monitor will oversee the progress of the trial and ensure it will be conducted in accordance with the protocol and Good Clinical Practice standards. AVD will be the main contact person for participating hospitals to report problems or to ask questions regarding the trial. Data entry will be performed by the data collectors.

#### Harms

The research team is committed to minimizing risks of harm and maximizing the benefits for potential participants. Participation in this study could be emotionally stressful as it involves coming into contact with themes that might confront them with their illness, prognosis and expectations for the future. We have put in place a procedure to identify and handle any signal of distress in the adolescent or the parents. The facilitators guiding the ACP conversations are experienced psychologists by background. At the beginning of the ACP conversation sessions, the facilitator will explicitly tell the adolescents and parents that they can always take a break when needed or stop participation whenever they want to. In addition, the facilitator will refer participants to the psychologists at the involved paediatric oncology ward if they notice they may need support. In that case, the facilitator will coordinate the contact with the psychologist from the ward. The facilitator will let the research team know and keep into touch with the psychologist to discuss potential continuation or termination of the family’s participation and will add this information to the structured diary. Contact details of the psychologists of the ward and of the research team are mentioned on the informational materials.

## Discussion

We currently lack robust evidence regarding the effectiveness of advance care planning in adolescents (pACP) who have cancer and their parents which applies randomised controlled trial designs. This study aims to improve, via a novel program, both parent-adolescent communication about advance care planning themes and improved self-efficacy in talking about ACP themes with each other, by contributing to a more positive attitude. The BOOST pACP program is a multicomponent program consisting of three ACP conversation sessions between an external facilitator and the adolescent and their parent(s). In this structured ACP approach, broader ACP themes such as experiences of the disease, preferences for communicating, decision-making by the adolescent and expectations for the future are discussed – hence considering ACP to be relevant from diagnosis onwards and not only targeting the end of life [6, 24, 47]. Normalizing communication about ACP themes between parents and the adolescent may lead to improved communication with healthcare professionals and trigger a feeling of empowerment of the adolescents in their own care plan and future wishes. The effectiveness, implementation, contextual factors and causal mechanisms will be evaluated in adolescents (age 10–18) and their parent(s), applying a randomised controlled trial design and embedded mixed-methods process evaluation.

Several potential limitations to the study design have to be considered. Firstly, the limited use of validated questionnaires to evaluate our selected endpoints; validated measures regarding the outcomes related to communication that are targeted with these kinds of interventions are often non-existent [19, 48]. Options are to either select validated measurement instruments that match the intervention’s goals to a lesser extent and are therefore less relevant, or adapt existing measures matching the desired goals or impact of the intervention, while compromising on validity. After an extensive search and multiple deliberations within the research team, we decided on slightly adapting the PACS to the context of ACP and to construct items based on the Theory of Planned Behaviour [49] regarding our secondary endpoints. We have performed a thorough cognitive testing of the measurement instrument with the target group. Secondly, given our strict inclusion criteria, the number of adolescents with cancer meeting our inclusion criteria might be lower than expected, complicating reaching sufficient sample size for this trial. However, we intend to mitigate this limitation by recruiting adolescents via four hospitals and relevant support organizations.

Mixed-methods results of this study can inform future implementation of ACP in this distinct population. If effective, the BOOST pACP model could represent an ACP approach for paediatric oncology that may also apply to other areas in paediatric care and other paediatric populations with distinct serious illnesses.

## Trial status

Recruitment started in January 2021 and is expected to be finalized in January 2023.

## Supplementary Information

**Additional file 1.** ‘Baseline questionnaires for adolescents, parents and paediatric oncologists’. These are the baseline questionnaires in Dutch language used for adolescents (the version for adolescents with two parents), parents and paediatric oncologists (including relevant clinical information about the adolescent).

**Additional file 2.** ‘Process evaluation methods based on UK MRC guidance on process evaluations of complex interventions (Moore et al., 2015)’. This table provides an overview of all process indicators that are evaluated in the process evaluation, including methods and at what timepoints.

## Data Availability

The datasets generated and/or analysed during the current study are available from the corresponding author on reasonable request.

## Abbreviations

ACP: Advance care planning
pACP: Paediatric advance care planning
PACS: Parent-adolescent communication scale
RCT: Randomised controlled trial
MRC: Medical Research Council
TIDieR: Template for intervention description and replication
AYA: Adolescents and young adults
SD: Standard deviation

## References

[1] Reedijk AMJ, Kremer LC, Visser O, Lemmens V, Pieters R, Coebergh JWW, Karim-Kos HE (2020). Increasing incidence of cancer and stage migration towards advanced disease in children and young adolescents in the Netherlands, 1990–2017. Eur J Cancer.

[2] Belgian Cancer Registry. Cancer in children and adolescents, Belgium 2004-2016, vol. 2016. p. 1–103.

[3] Kassam A, Widger K, Benini F. In: Wolfe J, Jones BL, Kreicbergs U, Jankovic M, editors. Palliative Care in Pediatric Oncology. Epidemiology of Suffering in Childhood Cancer: Springer International Publishing; 2018. p. 1–12.

[4] Basu S, Swil K (2018). Paediatric advance care planning: physician experience and education in initiating difficult discussions. J Paediatr Child Health.

[5] De Vleminck A, Houttekier D, Deliens L, Vander Stichele R, Pardon K. Development of a complex intervention to support the initiation of advance care planning by general practitioners in patients at risk of deteriorating or dying: a phase 0-1 study. BMC Palliat Care 2016;15(1):0–10, 17, DOI: 10.1186/s12904-016-0091-x. 10.1186/s12904-016-0091-x PMC4750213 26868650

[6] Rietjens JAC, Sudore RL, Connolly M, van Delden JJ, Drickamer MA, Droger M, van der Heide A, Heyland DK, Houttekier D, Janssen DJA, Orsi L, Payne S, Seymour J, Jox RJ, Korfage IJ, European Association for Palliative Care (2017). Definition and recommendations for advance care planning: an international consensus supported by the European Association for Palliative Care. Lancet Oncol.

[7] Jimenez G, Tan WS, Virk AK, Low CK, Car J, Ho AHY (2018). Overview of Systematic Reviews of Advance Care Planning: Summary of Evidence and Global Lessons. J Pain Symptom Manag.

[8] Yotani N, Kizawa Y, Shintaku H (2017). Advance care planning for adolescent patients with life-threatening neurological conditions: a survey of Japanese paediatric neurologists. BMJ Paediatr Open.

[9] Jacobs S, Perez J, Cheng YI, Sill A, Wang J, Lyon ME (2015). Adolescent end of life preferences and congruence with their parents’ preferences: results of a survey of adolescents with Cancer. Pediatr Blood Cancer.

[10] Lotz JD, Daxer M, Jox RJ, Borasio GD, Führer M (2017). “Hope for the best, prepare for the worst”: a qualitative interview study on parents’ needs and fears in pediatric advance care planning. Palliat Med.

[11] American Academy of Pediatrics, Committee on Bioethics and Committee on Hospital Care. Palliative Care for Children. J Pediatr. 2000;106(2):351-7. 10.1542/peds.106.2.351. 10920167

[12] Toce S, Collins MA. The FOOTPRINTS Model of Pediatric Palliative Care. J Palliat Med. 2003;6(6):989-1000. 10.1089/109662103322654910 14733693

[13] Hammes BJ, Klevan J, Kempf M, Williams MS (2005). Pediatric advance care planning. J Palliat Med.

[14] Lyon ME, Jacobs S, Briggs L, Cheng YI, Wang J (2013). Family-centered advance care planning for teens with cancer. JAMA Pediatr.

[15] Noyes J, Hastings RP, Lewis M, Hain R, Bennett V, Hobson L, et al. Planning ahead with children with life-limiting conditions and their families: development, implementation and evaluation of ‘My Choices’. BMC Palliat Care. 2013;12(1):1-17. 10.1186/1472-684X-12-5 PMC3579717 23384400

[16] Fahner J, Rietjens J, van der Heide A, Milota M, van Delden J, Kars M (2021). Evaluation showed that stakeholders valued the support provided by the implementing pediatric advance care planning toolkit. Acta Paediatr.

[17] Zadeh S, Pao M, Wiener L (2015). Opening End of Life Discussions: how to introduce Voicing My CHOiCES, and advance care planning guide for adolescents and young adults.

[18] DeCourcey DD, Partin L, Revette A, Bernacki R, Wolfe J (2021). Development of a Stakeholder Driven Serious Illness Communication Program for Advance Care Planning in Children, Adolescents, and Young Adults with Serious Illness. J Pediatr.

[19] Rietjens J, Korfage I, Taubert M (2020). Advance care planning: the future. BMJ Support Palliat Care.

[20] Weaver MS, Wiener L, Jacobs S, Bell CJ, Madrigal V, Mooney-Doyle K, Lyon ME (2021). Weaver et al’s response to Morrison: advance directives/care planning: clear, simple, and wrong. J Palliat Med.

[21] Aldridge J, Shimmon K, Miller M, Fraser LK, Wright B (2017). “I can’t tell my child they are dying”. Helping parents have conversations with their child. Arch Dis Child Educ Pract Ed.

[22] Hein K, Monz A, Daxer M, Heitkamp N, Knochel K, Jox R (2018). Challenges in pediatric advance care discussions between health care professionals and parents of children with a life-limiting condition: a qualitative pilot study. J Pain Symptom Manag.

[23] Marsac ML, Kindler C, Weiss D, Ragsdale L (2018). Let’s talk about it: supporting family communication during end-of-life Care of Pediatric Patients. J Palliat Med.

[24] Chan AW, Tetzlaff JM, Gøtzsche PC, Altman DG, Mann H, Berlin JA (2013). SPIRIT 2013 explanation and elaboration: guidance for protocols of clinical trials. BMJ..

[25] Sedgwick P (2013). What is a superiority trial?. BMJ..

[26] Moore GF, Audrey S, Barker M, Bond L, Bonell C, Hardeman W (2015). Process evaluation of complex interventions: Medical Research Council guidance. BMJ..

[27] Bleijenberg N, de Man-van Ginkel JM, Trappenburg JCA, Ettema RGA, Sino CG, Heim N (2018). Increasing value and reducing waste by optimizing the development of complex interventions: enriching the development phase of the Medical Research Council (MRC) framework. Int J Nurs Stud.

[28] O’Cathain A, Croot L, Duncan E, Rousseau N, Sworn K, Turner KM (2019). Guidance on how to develop complex interventions to improve health and healthcare. BMJ Open.

[29] Van Scoy LJ, Reading JM, Hopkins M, Smith B, Dillon J, Green MJ (2017). Community game day: using an end-of-life conversation game to encourage advance care planning. J Pain Symptom Manag.

[30] Kebbe M, Farmer A, Dyson MP, Scott SD, McHugh TLF, Lappa S (2019). Feasibility, user experiences, and preliminary effect of conversation cards for adolescents© on collaborative goal-setting and behavior change: protocol for a pilot randomized controlled trial. Pilot Feasibility Stud.

[31] Möller UO, Pranter C, Hagelin CL, Beck I, Malmström M, Fürst CJ, Rasmussen BH (2020). Using cards to facilitate conversations about wishes and priorities of patients in palliative care. J Hosp Palliat Nurs.

[32] Hoffmann TC, Glasziou PP, Boutron I, Milne R, Perera R, Moher D (2014). Better reporting of interventions: template for intervention description and replication (TIDieR) checklist and guide. BMJ..

[33] Elwyn G, Frosch D, Thomson R, Joseph-Williams N, Lloyd A, Kinnersley P, Cording E, Tomson D, Dodd C, Rollnick S, Edwards A, Barry M (2012). Shared decision making: a model for clinical practice. J Gen Intern Med.

[34] Haverman L, Grootenhuis MA, Raat H, van Rossum MAJ, van Dulmen-den Broeder E, Hoppenbrouwers K, Correia H, Cella D, Roorda LD, Terwee CB (2016). Dutch–Flemish translation of nine pediatric item banks from the patient-reported outcomes measurement information system (PROMIS)®. Qual Life Res.

[35] Mayoral K, Rajmil L, Murillo M, Garin O (2019). Measurement properties of the online EuroQol-5D-youth instrument in children and adolescents with type 1 diabetes mellitus : questionnaire study. J Med Internet Res.

[36] Elwyn G, Barr PJ, Grande SW, Thompson R, Walsh T, Ozanne EM (2013). Developing CollaboRATE: a fast and frugal patient-reported measure of shared decision making in clinical encounters. Patient Educ Couns.

[37] Keim MC, Lehmann V, Shultz EL, Winning AM, Rausch JR, Barrera M, Jo Gilmer M, Murphy LK, Vannatta KA, Compas BE, Gerhardt CA (2017). Parent-child communication and adjustment among children with advanced and non-advanced cancer in the first year following diagnosis or relapse. J Pediatr Psychol.

[38] Barnes HL, Olson DH (1985). Parent-adolescent and the Circumplex model. Soc Res Child Dev.

[39] Terwee CB, Roorda LD, De Vet HCW, Dekker J, Westhovens R, Van Leeuwen J (2014). Dutch-Flemish translation of 17 item banks from the patient-reported outcomes measurement information system (PROMIS). Qual Life Res.

[40] Lyon ME, Dallas RH, Hinds PS, Garvie A, Wilkins ML, Garcia A (2018). A randomized clinical trial of adolescents with HIV/AIDS: pediatric advance care planning.

[41] Ford CA, Mirman JH, García-España JF, Fisher Thiel MC, Friedrich E, Salek EC, Jaccard J (2019). Effect of primary care parent-targeted interventions on parent-adolescent communication about sexual behavior and alcohol use: a randomized clinical trial. JAMA Netw Open.

[42] Chu JTW, Wadham A, Jiang Y, Whittaker R, Stasiak K, Shepherd M, Bullen C (2019). Effect of MyTeen SMS-Based Mobile intervention for parents of adolescents: a randomized clinical trial. JAMA Netw Open.

[43] Sanders RA (2013). Adolescent psychosocial, social, and cognitive development. Pediatr Rev.

[44] Hartley CA, Somerville LH (2015). The neuroscience of adolescent decision-making. Curr Opin Behav Sci.

[45] Braun V, Clarke V (2006). Using thematic analysis in psychology. Qual Res Psychol.

[46] Nowell LS, Norris JM, White DE, Moules NJ (2017). Thematic analysis: striving to meet the trustworthiness criteria. Int J Qual Methods.

[47] Fahner JC (2020). Advance care planning in pediatrics [dissertation].

[48] Eklund R, Kreicbergs U, Alvariza A, Lövgren M (2018). The family talk intervention in palliative care: a study protocol. BMC Palliat Care.

[49] Bartholomew LK, Parcel GS, Kok G, Gottlieb NH, Fernandez ME (2011). Chapter 2: behavior-oriented theories used in health promotion. Planning health promotion programs an intervention mapping approach.

