# Supplementary material for: Advance care planning for adolescents with cancer and their parents: study protocol of the BOOST pACP multi-centre randomised controlled trial and process evaluation
Source: BMC Pediatr. 2021 Sep 1;21:376. doi: 10.1186/s12887-021-02841-7 (PMC8408307; doi:10.1186/s12887-021-02841-7)
Supplement: Supplementary file 1 — Additional file 1. ‘Baseline questionnaires for adolescents, parents and paediatric oncologists’. These are the baseline questionnaires in Dutch language used for adolescents (the version for adolescents with two parents), parents and paediatric oncologists (including relevant clinical information about the adolescent). [file 12887_2021_2841_MOESM1_ESM.pdf]

T0 (2 ouders)

Deelnemerscode (in te vullen door onderzoeker, bv AN05J-T1):

Additional file 1: Baseline questionnaires for adolescents, parents and paediatric oncologists

BOOST

Samen Praten over Zorg

Vragenlijst voor jongeren

BOOST: een studie rond vroegtijdige zorgplanning in de kinderoncologie
Vragenlijst voor jongeren

Uitleg bij deze vragenlijst

Bedankt dat je meewerkt aan ons onderzoek!

Waar gaan de vragen in de vragenlijst over?
Graag komen wij meer te weten over in hoeverre jij hebt nagedacht en gesproken over wat jij belangrijk vindt, als het gaat om de zorg en behandeling. Iemand zal deze vragenlijst met jou overlopen. De vragenlijst gaat over verschillende dingen.

- Deel 1. Praat jij met je ouders of voogd over je zorg en behandeling? En wat vind je van die gesprekken?
- Deel 2. Praat je over je zorg en behandeling met je kinderoncoloog (je arts in het ziekenhuis)?
- Deel 3. Hoe heb je je gevoeld in de afgelopen 7 dagen en hoe voel je je vandaag?
- Deel 4. Wie ben jij?
- Deel 5. Heb je nog opmerkingen?

Hoe vul ik de vragenlijst in?
Iemand zal deze vragenlijst met jou overlopen. Het invullen duurt ongeveer 50 minuten. Je kunt ervoor kiezen de vragenlijst samen in te vullen op papier of digitaal via https://tinyurl.com/BoostStudie-J-base2.

Enkele instructies voor je van start gaat:

1. De meeste vragen kan je beantwoorden door één van de vakjes aan te vinken: .
   Indien je meer dan één antwoord aan kunt duiden, staat dit bij de vraag vermeld. Bij de open vragen kun je zelf tegen de persoon met wie je de vragenlijst invult zeggen wat je wilt invullen.
2. Het is belangrijk voor het onderzoek dat je alle vragen in de vragenlijst beantwoordt. Of je nu wel of niet over je zorg en behandeling hebt nagedacht of gepraat, zolang je aankruist wat voor jou geldt is het helemaal goed. Dan help je ons het beste met je antwoorden.
3. De persoon die samen met jou de vragenlijst invult kan je helpen als je vragen hebt. Je kan haar altijd vragen stellen.

Wie krijgt mijn antwoorden?
De antwoorden worden alleen bezorgd aan de onderzoekers die dit onderzoek uitvoeren. We geven jouw antwoorden een code uit letters en cijfers zonder jouw naam, zodat niemand kan weten van wie de antwoorden komen.

Vragen?
Contacteer Anne van Driessche (uitvoerend onderzoeker)
tel: +32(0)456302963
e-mail: Anne.van.Driessche@vub.be

Deel 1: Jouw zorg en behandeling

Welke zorg en behandeling tegen kanker heb je nodig? Misschien is daar met jou en je ouders al veel over gepraat. Maar wat vind jij zelf belangrijk? En met wie praat jij daarover? Daarover gaat dit deel van de vragenlijst.

We hebben het niet alleen over jouw medische behandeling die je krijgt van zorgverleners in het ziekenhuis (zoals de verpleegkundige, arts of psycholoog). We bedoelen met **zorg** ook de hulp of steun die je krijgt van andere mensen, zoals van je ouders, vrienden of familie.

Wil je alsjeblieft ook de datum van vandaag invullen?

1. **De datum vandaag is:** ________ (dag)/________ (maand)/__________________(jaar)

2. Wie van jouw ouders of verzorgers is het meest aanwezig als je afspraken hebt in het ziekenhuis?

- ☐ Mijn moeder
- ☐ Mijn vader
- ☐ Beiden evenveel
- ☐ Iemand anders (vul in): ____________

Ga verder op de volgende pagina

Deel 1a: Over praten met je moeder

Geef aan de hand van onderstaande schaal aan in welke mate je het eens of oneens bent met ELK van de volgende zinnen.

3. De zinnen gaan over in hoeverre jij met je moeder kan praten over wat jij belangrijk vindt in jouw zorg en behandeling.	Helemaal oneens	Oneens	Niet eens en niet oneens	Eens	Helemaal eens
a. Ik kan mijn mening bespreken met mijn moeder zonder dat ik het gevoel heb dat ik me in moet houden of dat ik me ongemakkelijk voel.	☐	☐	☐	☐	☐
b. Ik vind het soms moeilijk om alles te geloven wat mijn moeder me vertelt.	☐	☐	☐	☐	☐
c. Mijn moeder luistert altijd goed.	☐	☐	☐	☐	☐
d. Ik ben soms bang om iets wat ik graag wil aan mijn moeder te vragen.	☐	☐	☐	☐	☐
e. Mijn moeder zegt soms dingen tegen mij die beter niet gezegd zouden worden.	☐	☐	☐	☐	☐
f. Mijn moeder weet hoe ik me voel zonder iets te vragen.	☐	☐	☐	☐	☐
g. Ik ben heel tevreden over de manier waarop mijn moeder en ik met elkaar praten.	☐	☐	☐	☐	☐
h. Als ik in de problemen zou zitten, zou ik het aan mijn moeder kunnen vertellen.	☐	☐	☐	☐	☐
i. Ik toon openlijk dat ik mijn moeder graag zie.	☐	☐	☐	☐	☐
j. Als mijn moeder en ik een probleem hebben, dan praat ik niet meer tegen haar.	☐	☐	☐	☐	☐
k. Ik ben voorzichtig in wat ik mijn moeder zeg.	☐	☐	☐	☐	☐
l. Als ik met mijn moeder praat, zeg ik soms dingen die ik beter niet zou zeggen.	☐	☐	☐	☐	☐
m. Als ik vragen stel, krijg ik een eerlijk antwoord van mijn moeder.	☐	☐	☐	☐	☐
n. Mijn moeder probeert mijn mening te begrijpen.	☐	☐	☐	☐	☐
o. Er zijn onderwerpen waarover ik liever niet praat met mijn moeder.	☐	☐	☐	☐	☐
p. Ik vind het gemakkelijk om problemen te bespreken met mijn moeder.	☐	☐	☐	☐	☐
q. Het is heel gemakkelijk voor mij om al mijn echte gevoelens uit te drukken bij mijn moeder.	☐	☐	☐	☐	☐
r. Mijn moeder zeurt/werkt op mijn zenuwen.	☐	☐	☐	☐	☐
s. Mijn moeder beledigt me soms als zij boos op me is.	☐	☐	☐	☐	☐

4. De volgende zinnen gaan over praten met je moeder over wat je belangrijk vindt in je zorg en behandeling. Hoezeer ben je het eens met elke zin? Kruis aan.	Helemaal oneens	Oneens	Niet eens en niet oneens	Eens	Helemaal eens
a. Ik vind het belangrijk om met mijn moeder te praten over wat ik belangrijk vind in mijn zorg en behandeling.	☐	☐	☐	☐	☐
b. Ik vind het oké als mijn moeder wil praten over wat ik belangrijk vind in mijn zorg en behandeling.	☐	☐	☐	☐	☐
c. Ik ben bang dat ik mijn moeder verdrietig maak als ik praat over wat ik belangrijk vind in mijn zorg en behandeling.	☐	☐	☐	☐	☐
d. Ik weet te weinig over mijn ziekte of behandeling om met mijn moeder te praten over wat ik belangrijk vind.	☐	☐	☐	☐	☐
e. Ik denk dat mijn moeder mij te jong vindt om mee te praten over wat ik belangrijk vind in mijn zorg en behandeling.	☐	☐	☐	☐	☐
f. Ik denk dat er toch niets verandert als ik praat met mijn moeder over wat ik belangrijk vind in mijn zorg en behandeling.	☐	☐	☐	☐	☐
Denk aan de zorg die je **nu** krijgt.<br>g. Ik vind het makkelijk om met mijn moeder te praten over wat ik belangrijk vind in mijn zorg en behandeling.	☐	☐	☐	☐	☐
Denk aan de zorg die je **later** kan krijgen.<br>h. Ik vind het makkelijk om met mijn moeder te praten over wat ik belangrijk vind in mijn zorg en behandeling.	☐	☐	☐	☐	☐

5. Als ik dat zou willen, zou het mij goed lukken om met mijn moeder te praten over…	Helemaal oneens	Oneens	Niet eens en niet oneens	Eens	Helemaal eens
a. wat ik belangrijk vind in mijn leven	☐	☐	☐	☐	☐
b. hoe ik me voel door mijn ziekte en behandeling	☐	☐	☐	☐	☐
c. waarover ik veel nadenk of pieker	☐	☐	☐	☐	☐
d. waarvoor ik bang ben	☐	☐	☐	☐	☐
e. waarop ik hoop	☐	☐	☐	☐	☐
f. wat ik verwacht van de toekomst	☐	☐	☐	☐	☐
g. het wel of niet starten van een nieuwe behandeling, als de behandeling die ik nu krijg niet meer zou werken	☐	☐	☐	☐	☐
h. het stoppen van de behandeling, als die niet meer zou werken	☐	☐	☐	☐	☐
i. de kans dat ik zou sterven	☐	☐	☐	☐	☐

6. Ik heb in de afgelopen maand met mijn moeder gepraat over…	Ja	Nee
a. wat ik belangrijk vind in mijn leven	☐	☐
b. hoe ik me voel door mijn ziekte en behandeling	☐	☐
c. waarover ik veel nadenk of pieker	☐	☐
d. waarvoor ik bang ben	☐	☐
e. waarop ik hoop	☐	☐
f. wat ik verwacht van de toekomst	☐	☐
g. het wel of niet starten van een nieuwe behandeling, als de behandeling die ik nu krijg niet meer zou werken	☐	☐
h. het stoppen van de behandeling, als die niet meer zou werken	☐	☐
i. de kans dat ik zou sterven	☐	☐

7. Ik wil binnenkort met mijn moeder praten over…	Ja	Nee
a. wat ik belangrijk vind in mijn leven	☐	☐
b. hoe ik me voel door mijn ziekte en behandeling	☐	☐
c. waarover ik veel nadenk of pieker	☐	☐
d. waarvoor ik bang ben	☐	☐
e. waarop ik hoop	☐	☐
f. wat ik verwacht van de toekomst	☐	☐
g. het wel of niet starten van een nieuwe behandeling, als de behandeling die ik nu krijg niet meer zou werken	☐	☐
h. het stoppen van de behandeling, als die niet meer zou werken	☐	☐
i. de kans dat ik zou sterven	☐	☐

Ga verder op de volgende pagina

Deel 1b: Over praten met je vader

Geef aan de hand van onderstaande schaal aan in welke mate je het eens of oneens bent met ELK van de volgende zinnen.

8. De zinnen gaan over in hoeverre jij met je vader kan praten over wat jij belangrijk vindt in jouw zorg en behandeling.	Helemaal oneens	Oneens	Niet eens en niet oneens	Eens	Helemaal eens
a. Ik kan mijn mening bespreken met mijn vader zonder dat ik het gevoel heb dat ik me in moet houden of dat ik me ongemakkelijk voel.	☐	☐	☐	☐	☐
b. Ik vind het soms moeilijk om alles te geloven wat mijn vader me vertelt.	☐	☐	☐	☐	☐
c. Mijn vader luistert altijd goed.	☐	☐	☐	☐	☐
d. Ik ben soms bang om iets wat ik graag wil aan mijn vader te vragen.	☐	☐	☐	☐	☐
e. Mijn vader zegt soms dingen tegen mij die beter niet gezegd zouden worden.	☐	☐	☐	☐	☐
f. Mijn vader weet hoe ik me voel zonder iets te vragen.	☐	☐	☐	☐	☐
g. Ik ben heel tevreden over de manier waarop mijn vader en ik met elkaar praten.	☐	☐	☐	☐	☐
h. Als ik in de problemen zou zitten, zou ik het aan mijn vader kunnen vertellen.	☐	☐	☐	☐	☐
i. Ik toon openlijk dat ik mijn vader graag zie.	☐	☐	☐	☐	☐
j. Als mijn vader en ik een probleem hebben, dan praat ik niet meer tegen hem.	☐	☐	☐	☐	☐
k. Ik ben voorzichtig in wat ik mijn vader zeg.	☐	☐	☐	☐	☐
l. Als ik met mijn vader praat, zeg ik soms dingen die ik beter niet zou zeggen.	☐	☐	☐	☐	☐
m. Als ik vragen stel, krijg ik een eerlijk antwoord van mijn vader.	☐	☐	☐	☐	☐
n. Mijn vader probeert mijn mening te begrijpen.	☐	☐	☐	☐	☐
o. Er zijn onderwerpen waarover ik liever niet praat met mijn vader.	☐	☐	☐	☐	☐
p. Ik vind het gemakkelijk om problemen te bespreken met mijn vader.	☐	☐	☐	☐	☐
q. Het is heel gemakkelijk voor mij om al mijn echte gevoelens uit te drukken bij mijn vader.	☐	☐	☐	☐	☐
r. Mijn vader zeurt/werkt op mijn zenuwen.	☐	☐	☐	☐	☐
s. Mijn vader beledigt me soms als hij boos op me is.	☐	☐	☐	☐	☐

9. De volgende zinnen gaan over praten met je vader over wat je belangrijk vindt in je zorg en behandeling. Hoezeer ben je het eens met elke zin? Kruis aan.	Helemaal oneens	Oneens	Niet eens en niet oneens	Eens	Helemaal eens
a. Ik vind het belangrijk om met mijn vader te praten over wat ik belangrijk vind in mijn zorg en behandeling.	☐	☐	☐	☐	☐
b. Ik vind het oké als mijn vader wil praten over wat ik belangrijk vind in mijn zorg en behandeling.	☐	☐	☐	☐	☐
c. Ik ben bang dat ik mijn vader verdrietig maak als ik praat over wat ik belangrijk vind in mijn zorg en behandeling.	☐	☐	☐	☐	☐
d. Ik weet te weinig over mijn ziekte of behandeling om met mijn vader te praten over wat ik belangrijk vind.	☐	☐	☐	☐	☐
e. Ik denk dat mijn vader mij te jong vindt om mee te praten over wat ik belangrijk vind in mijn zorg en behandeling.	☐	☐	☐	☐	☐
f. Ik denk dat er toch niets verandert als ik praat met mijn vader over wat ik belangrijk vind in mijn zorg en behandeling.	☐	☐	☐	☐	☐
Denk aan de zorg die je **nu** krijgt.<br>g. Ik vind het makkelijk om met mijn vader te praten over wat ik belangrijk vind in mijn zorg en behandeling.	☐	☐	☐	☐	☐
Denk aan de zorg die je **later** kan krijgen.<br>h. Ik vind het makkelijk om met mijn vader te praten over wat ik belangrijk vind in mijn zorg en behandeling.	☐	☐	☐	☐	☐

10. Als ik dat zou willen, zou het mij goed lukken om met mijn vader te praten over...	Helemaal oneens	Oneens	Niet eens en niet oneens	Eens	Helemaal eens
a. wat ik belangrijk vind in mijn leven	☐	☐	☐	☐	☐
b. hoe ik me voel door mijn ziekte en behandeling	☐	☐	☐	☐	☐
c. waarover ik veel nadenk of pieker	☐	☐	☐	☐	☐
d. waarvoor ik bang ben	☐	☐	☐	☐	☐
e. waarop ik hoop	☐	☐	☐	☐	☐
f. wat ik verwacht van de toekomst	☐	☐	☐	☐	☐
g. het wel of niet starten van een nieuwe behandeling, als de behandeling die ik nu krijg niet meer zou werken	☐	☐	☐	☐	☐
h. het stoppen van de behandeling, als die niet meer zou werken	☐	☐	☐	☐	☐
i. de kans dat ik zou sterven	☐	☐	☐	☐	☐

11. Ik heb in de afgelopen maand met mijn vader gepraat over…:	Ja	Nee
a. wat ik belangrijk vind in mijn leven	☐	☐
b. hoe ik me voel door mijn ziekte en behandeling	☐	☐
c. waarover ik veel nadenk of pieker	☐	☐
d. waarvoor ik bang ben	☐	☐
e. waarop ik hoop	☐	☐
f. wat ik verwacht van de toekomst	☐	☐
g. het wel of niet starten van een nieuwe behandeling, als de behandeling die ik nu krijg niet meer zou werken	☐	☐
h. het stoppen van de behandeling, als die niet meer zou werken	☐	☐
i. de kans dat ik zou sterven	☐	☐

12. Ik wil binnenkort met mijn vader praten over…:	Ja	Nee
a. wat ik belangrijk vind in mijn leven	☐	☐
b. hoe ik me voel door mijn ziekte en behandeling	☐	☐
c. waarover ik veel nadenk of pieker	☐	☐
d. waarvoor ik bang ben	☐	☐
e. waarop ik hoop	☐	☐
f. wat ik verwacht van de toekomst	☐	☐
g. het wel of niet starten van een nieuwe behandeling, als de behandeling die ik nu krijg niet meer zou werken	☐	☐
h. het stoppen van de behandeling, als die niet meer zou werken	☐	☐
i. de kans dat ik zou sterven	☐	☐

Ga verder op de volgende pagina

Deel 2: Praten met een kinderoncoloog uit het ziekenhuis

13. Heb je in de afgelopen maand nog een afspraak gehad in het ziekenhuis bij de kinderoncoloog?

- ☐ Ja, één afspraak
- ☐ Ja, meerdere afspraken
- ☐ Nee
- ☐ Ik weet het niet meer

14. Ik heb in de afgelopen maand met een kinderoncoloog gepraat over…:	Ja	Nee
a. wat ik belangrijk vind in mijn leven	☐	☐
b. hoe ik me voel door mijn ziekte en behandeling	☐	☐
c. waarover ik veel nadenk of pieker	☐	☐
d. waarvoor ik bang ben	☐	☐
e. waarop ik hoop	☐	☐
f. wat ik verwacht van de toekomst	☐	☐
g. het wel of niet starten van een nieuwe behandeling, als de behandeling die ik nu krijg niet meer zou werken	☐	☐
h. het stoppen van de behandeling, als die niet meer zou werken	☐	☐
i. de kans dat ik zou sterven	☐	☐

15. Ik wil binnenkort met een kinderoncoloog praten over…:	Ja	Nee
a. wat ik belangrijk vind in mijn leven	☐	☐
b. hoe ik me voel door mijn ziekte en behandeling	☐	☐
c. waarover ik veel nadenk of pieker	☐	☐
d. waarvoor ik bang ben	☐	☐
e. waarop ik hoop	☐	☐
f. wat ik verwacht van de toekomst	☐	☐
g. het wel of niet starten van een nieuwe behandeling, als de behandeling die ik nu krijg niet meer zou werken	☐	☐
h. het stoppen van de behandeling, als die niet meer zou werken	☐	☐
i. de kans dat ik zou sterven	☐	☐

Deel 3: Hoe heb je je gevoeld in de afgelopen 7 dagen en hoe voel je je vandaag?

16. Deze vragen gaan over hoe jij **de afgelopen 7 dagen** gevoeld hebt.	Nooit	Bijna nooit	Soms	Vaak	Bijna altijd
a. Ik had het gevoel dat er iets verschrikkelijks zou kunnen gebeuren.	☐	☐	☐	☐	☐
b. Ik voelde me zenuwachtig.	☐	☐	☐	☐	☐
c. Ik voelde me bang.	☐	☐	☐	☐	☐
d. Ik maakte me zorgen.	☐	☐	☐	☐	☐
e. Ik maakte me zorgen als ik thuis was.	☐	☐	☐	☐	☐
f. Ik werd echt gauw bang.	☐	☐	☐	☐	☐
g. Ik maakte me zorgen over wat er met me zou kunnen gebeuren.	☐	☐	☐	☐	☐
h. Ik maakte me zorgen als ik 's avonds naar bed ging.	☐	☐	☐	☐	☐
i. Ik voelde me gelukkig.	☐	☐	☐	☐	☐
j. Ik voelde me opgelucht.	☐	☐	☐	☐	☐
k. Ik voelde me mentaal sterk.	☐	☐	☐	☐	☐

**17. Zet bij iedere groep hieronder een kruisje in het ENE hokje dat het best past bij je eigen gezondheid VANDAAG**	Nee	Een beetje	Veel
a. Ik heb problemen met rondwandelen.	☐	☐	☐
b. Ik heb problemen met wassen of aankleden.	☐	☐	☐
c. Ik heb problemen met mijn dagelijkse activiteiten (bijvoorbeeld naar school gaan, hobby's, sporten, spelen, dingen doen met familie of vrienden).	☐	☐	☐
d. Ik heb pijn of andere ongemakken.	☐	☐	☐
e. Ik ben bezorgd, verdrietig of ongelukkig.	☐	☐	☐

Ga verder op de volgende pagina

18.

- We willen graag weten hoe goed of slecht je gezondheid VANDAAG is.
- Daarvoor hebben we deze genummerde lijn van 0 tot en met 100 getekend.
- 100 geeft de <u>beste</u> gezondheid aan die je je kunt voorstellen.
  0 geeft de <u>slechtste</u> gezondheid aan die je je kunt voorstellen.
- Plaats een X op de lijn om aan te geven hoe goed of slecht je gezondheid VANDAAG is

De beste gezondheid die je je kunt voorstellen

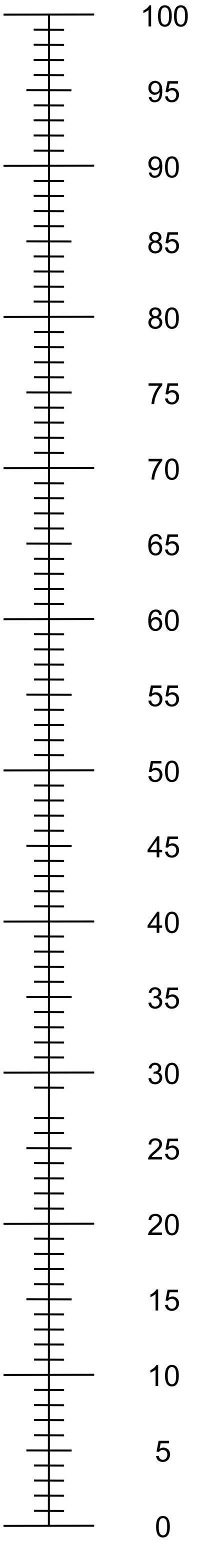

De slechtste gezondheid die je je kunt voorstellen

Deel 4: Wie ben jij?

19.

a. Ik ben een

- ☐ Jongen/ man
- ☐ Meisje/ vrouw
- ☐ Anders

b. Dit is mijn geboortedatum: ________________ (dag – maand – jaar)

c. Ik ben in ______________________________ geboren
(. geef jouw geboorteland bijvoorbeeld: België, Marokko, Spanje)

Deel 5: Heb je nog opmerkingen?

20. Schrijf het hier:

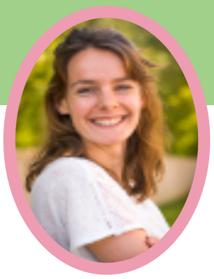

Onderzoeker
Anne van Driessche

DANK JE!

Vragen of opmerkingen? Stuur een mailtje naar
Anne.van.Driessche@vub.be of bel op +32(0)456302963

BOOST
Samen Praten over Zorg

Vragenlijst voor ouders

(TO)

BOOST: een studie rond vroegtijdige zorgplanning in de kinderoncologie

Vragenlijst voor ouders

Uitleg bij deze vragenlijst

Bedankt dat u meewerkt aan ons onderzoek!

Waarover gaat het onderzoek?

Dit onderzoek wordt uitgevoerd door onderzoekers van de Vrije Universiteit Brussel (VUB) en Universiteit Gent (UGent) en is gefinancieerd door het Fonds Wetenschappelijk Onderzoek (FWO) en het Kinderkankerfonds. Het doel van het project is om in de toekomst de zorg beter af te stemmen op de wensen en noden van adolescenten en ouders.

Waar gaan de vragen in de vragenlijst over?

Graag komen wij meer te weten over in hoeverre u met uw kind praat over welke zorg en behandeling uw kind wilt. De vragenlijst gaat over verschillende dingen.

Deel 1. Wat uw kind belangrijk vindt in zijn of haar zorg of behandeling
Deel 2. Over uw laatste contact met een kinderoncoloog
Deel 3. Hoe heeft u zich de afgelopen 7 dagen gevoeld?
Deel 4. Demografische gegevens
Deel 5. Heeft u nog opmerkingen?

Hoe vul ik de vragenlijst in?

Het invullen duurt ongeveer 30 - 40 minuten. U kunt ervoor kiezen de vragenlijst in te vullen op papier of digitaal via https://tinyurl.com/BoostStudie-ouderbase.

Enkele instructies voor u van start gaat:

4. De meeste vragen kunt u beantwoorden door één van de vakjes aan te vinken: ☑. Indien u meer dan één antwoord aan kunt duiden, staat dit bij de vraag vermeld. Bij de open vragen kunt u zelf iets neerschrijven.
5. Het is belangrijk voor het onderzoek dat u alle vragen in de vragenlijst beantwoordt. Goede of foute antwoorden zijn er niet. Communicatie tussen kind en ouder(s) is uniek voor elk gezin en loopt bij iedereen anders. Wij willen enkel inzicht krijgen in hoe jullie communicatie verloopt. De vragen in deze vragenlijst zeggen niets over de kwaliteit van de communicatie of de kwaliteit van de relatie tussen jullie beide. Soms kan het moeilijk zijn om een precies antwoord te geven. Vink dan het vakje aan dat zo dicht mogelijk aanleunt bij uw antwoord.

Vertrouweljkheid van uw gegevens

De gegevens die in het kader van uw deelname worden verzameld zijn vertrouwelijk, in overeenstemming met de Belgische Wetgeving op de bescherming van de persoonljke levenssfeer.

Vragen?

Contacteer Anne van Driessche (uitvoerend onderzoeker)
tel: +32(0)456302963
e-mail: Anne.van.Driessche@vub.be

Deel 1: Wat uw kind belangrijk vindt in zijn of haar zorg en behandeling

Welke zorg en behandeling wil uw kind? En praat u daarover met hem of haar? Dat onderzoeken we met deze vragenlijst.

In deze vragenlijst hebben we het niet alleen over de medische behandeling tegen kanker. We bedoelen met zorg **ook** verzorging en ondersteuning die uw kind krijgt of wil krijgen. Bijvoorbeeld van u als ouder of wettelijke voogd.

1. De datum vandaag is: ________ (dag)/________ (maand)/__________________(jaar)

**2. Deze vragen gaan over praten met uw kind over wat hij of zij belangrijk vindt in de zorg en behandeling. Geef aan in hoeverre u het eens of oneens bent met de volgende uitspraken**	Helemaal oneens	Oneens	Niet eens en niet oneens	Eens	Helemaal eens
i. Ik vind het belangrijk om met mijn kind te praten over wat hij of zij belangrijk vindt in de zorg en behandeling.	☐	☐	☐	☐	☐
j. Ik vind het moeilijk om te weten te komen wat mijn kind belangrijk vindt in de zorg en behandeling.	☐	☐	☐	☐	☐
k. Ik vraag mijn kind nadrukkelijk wat hij of zij belangrijk vindt in de zorg en behandeling.	☐	☐	☐	☐	☐
l. Ik ben bang dat ik mijn kind verdrietig maak als ik praat over wat hij of zij belangrijk vindt in de zorg en behandeling.	☐	☐	☐	☐	☐
m. Ik vind dat mijn kind nog niet mee kan beslissen over de zorg en behandeling.	☐	☐	☐	☐	☐
n. Als mijn kind wil praten over de zorg en behandeling, dan sta ik daarvoor open.	☐	☐	☐	☐	☐
o. Ik praat gemakkelijk met mijn kind over wat hij of zij belangrijk vindt in de zorg en behandeling.	☐	☐	☐	☐	☐

h. Indien u dat wenst, kunt u hier eventuele toelichting schrijven.

3. Hoezeer lukken deze zaken u?	Helemaal oneens	Oneens	Niet eens en niet oneens	Eens	Helemaal eens
a. Het lukt mij goed om te praten met mijn kind, als hij of zij anders denkt over de zorg of behandeling dan ik.	☐	☐	☐	☐	☐
b. Het lukt mij goed om te weten te komen wat mijn kind écht wil in de zorg of behandeling.	☐	☐	☐	☐	☐
c. Het lukt mij goed om mijn kind het gevoel te geven dat hij of zij over alles kan praten met mij.	☐	☐	☐	☐	☐
d. Het lukt mij goed om mijn kind tijdens een consultatie zelf te laten praten met een arts over wat hij of zij belangrijk vindt in de zorg en behandeling.	☐	☐	☐	☐	☐
e. Het lukt mij goed om mijn kind te stimuleren om te zeggen wat hij of zij belangrijk vindt in de zorg en behandeling.	☐	☐	☐	☐	☐
f. Het lukt mij goed om met mijn kind tot gezamenlijke besluiten te komen wat betreft de zorg en behandeling.	☐	☐	☐	☐	☐

g. Indien u dat wenst, kunt u hier eventuele toelichting schrijven.

4. Het zou mij goed lukken om een gesprek te hebben met mijn kind als hij of zij begint over…:	Helemaal oneens	Oneens	Niet eens en niet oneens	Eens	Helemaal eens
j. wat mijn kind belangrijk vindt in het leven	☐	☐	☐	☐	☐
k. hoe mijn kind zich voelt door de ziekte en behandeling	☐	☐	☐	☐	☐
l. waarover mijn kind piekert	☐	☐	☐	☐	☐
m. waarvoor mijn kind bang is	☐	☐	☐	☐	☐
n. waarop mijn kind hoopt	☐	☐	☐	☐	☐
o. wat mijn kind verwacht van de toekomst	☐	☐	☐	☐	☐
p. het wel of niet starten van een nieuwe behandeling, als de behandeling die mijn kind nu krijgt niet meer zou werken	☐	☐	☐	☐	☐
q. het stoppen van de behandeling, als de behandeling die mijn kind nu krijgt niet meer zou werken	☐	☐	☐	☐	☐
r. of er een kans bestaat dat mijn kind zou sterven aan de ziekte	☐	☐	☐	☐	☐

j. Indien u dat wenst, kunt u hier eventuele toelichting schrijven.

5. Als ik daar nood aan heb, zou het mij goed lukken om zelf te beginnen praten met mijn kind over…:	Helemaal oneens	Oneens	Niet eens en niet oneens	Eens	Helemaal eens
a. wat mijn kind belangrijk vindt in het leven	☐	☐	☐	☐	☐
b. hoe mijn kind zich voelt door de ziekte en behandeling	☐	☐	☐	☐	☐
c. waarover mijn kind piekert	☐	☐	☐	☐	☐
d. waarvoor mijn kind bang is	☐	☐	☐	☐	☐
e. waarop mijn kind hoopt	☐	☐	☐	☐	☐
f. wat mijn kind verwacht van de toekomst	☐	☐	☐	☐	☐
g. het wel of niet starten van een nieuwe behandeling, als de behandeling die mijn kind nu krijgt niet meer zou werken	☐	☐	☐	☐	☐
h. het stoppen van de behandeling, als de behandeling die mijn kind nu krijgt niet meer zou werken	☐	☐	☐	☐	☐
i. of er een kans bestaat dat mijn kind zou sterven aan de ziekte	☐	☐	☐	☐	☐

j. Indien u dat wenst, kunt u hier eventuele toelichting schrijven.

6. Ik heb de afgelopen 30 dagen met mijn kind gepraat over…:	Ja	Nee
a. wat mijn kind belangrijk vindt in het leven	☐	☐
b. hoe mijn kind zich voelt door de ziekte en behandeling	☐	☐
c. waarover mijn kind piekert	☐	☐
d. waarvoor mijn kind bang is	☐	☐
e. waarop mijn kind hoopt	☐	☐
f. wat mijn kind verwacht van de toekomst	☐	☐
g. het wel of niet starten van een nieuwe behandeling, als de behandeling die mijn kind nu krijgt niet meer zou werken	☐	☐
h. het stoppen van de behandeling, als de behandeling die mijn kind nu krijgt niet meer zou werken	☐	☐
i. of er een kans bestaat dat mijn kind zou sterven aan de ziekte	☐	☐

j. Indien u dat wenst, kunt u hier eventuele toelichting schrijven.

7. Ik ben van plan binnenkort te praten met mijn kind over...:	Ja	Nee
a. wat mijn kind belangrijk vindt in het leven	☐	☐
b. hoe mijn kind zich voelt door de ziekte en behandeling	☐	☐
c. waarover mijn kind piekert	☐	☐
d. waarvoor mijn kind bang is	☐	☐
e. waarop mijn kind hoopt	☐	☐
f. wat mijn kind verwacht van de toekomst	☐	☐
g. het wel of niet starten van een nieuwe behandeling, als de behandeling die mijn kind nu krijgt niet meer zou werken	☐	☐
h. het stoppen van de behandeling, als de behandeling die mijn kind nu krijgt niet meer zou werken	☐	☐
i. of er een kans bestaat dat mijn kind zou sterven aan de ziekte	☐	☐

j. Indien u dat wenst, kunt u hier eventuele toelichting schrijven.

Ga verder op de volgende pagina

Deel 2: Over uw laatste contact met een kinderoncoloog

8. Heeft u in de afgelopen 30 dagen nog een afspraak gehad in het ziekenhuis bij de kinderoncoloog?

- ☐ Ja, één afspraak
- ☐ Ja, meerdere afspraken
- ☐ Nee
- ☐ Ik weet het niet meer

Als u terugdenkt aan het laatste bezoek aan uw kinderoncoloog…
Omcirkel een cijfer aan dat voor u het beste past.

9. Hoeveel moeite is er gedaan om u te helpen de gezondheidssituatie van uw kind te begrijpen? *(omcirkel)*

0	1	2	3	4	5	6	7	8	9
Helemaal geen moeite gedaan									Alle mogelijke moeite gedaan

10. Hoeveel moeite is er gedaan om te luisteren naar de dingen die voor u belangrijk zijn als het gaat om de gezondheidssituatie van uw kind? *(omcirkel)*

0	1	2	3	4	5	6	7	8	9
Helemaal geen moeite gedaan									Alle mogelijke moeite gedaan

11. Hoeveel moeite is er gedaan om de dingen die voor u belangrijk zijn mee te nemen bij het kiezen van een volgende stap? *(omcirkel)*

0	1	2	3	4	5	6	7	8	9
Helemaal geen moeite gedaan									Alle mogelijke moeite gedaan

Deel 3: Hoe heeft u zich de afgelopen 7 dagen gevoeld?

12. Deze vragen gaan over hoe u zich **de afgelopen 7 dagen** gevoeld heeft.	Nooit	Bijna nooit	Soms	Vaak	Bijna altijd
a. Ik voelde me angstig.	☐	☐	☐	☐	☐
b. Ik voelde me angstig en bezorgd.	☐	☐	☐	☐	☐
c. Ik maakte me zorgen.	☐	☐	☐	☐	☐
d. Ik vond het moeilijk om me op iets anders dan mijn angst en mijn bezorgdheid te concentreren.	☐	☐	☐	☐	☐
e. Ik voelde me zenuwachtig.	☐	☐	☐	☐	☐
f. Ik voelde me slecht op mijn gemak.	☐	☐	☐	☐	☐
g. Ik voelde me gespannen.	☐	☐	☐	☐	☐
h. Ik voelde me gelukkig.	☐	☐	☐	☐	☐
i. Ik voelde me opgelucht.	☐	☐	☐	☐	☐
j. Ik voelde me mentaal sterk.	☐	☐	☐	☐	☐

Deel 4: Demografische gegevens

13. Vul hier uw persoonlijke gegevens in:	
a. **Wat is uw geslacht?**	☐ Man<br>☐ Vrouw<br>☐ Anders
b. **Wat is het geslacht van uw kind?**	☐ Man<br>☐ Vrouw<br>☐ Anders
c. **Wat is uw geboortedatum?**	_______/________/______________(dd/mm/jjjj)
d. **Wat is de geboortedatum van uw kind?**	_______/________/______________(dd/mm/jjjj)
e. **Wat is uw hoogst behaalde diploma?**	☐ geen<br>☐ lager onderwijs<br>☐ lager middelbaar<br>☐ hoger middelbaar<br>☐ graduaat (A2)<br>☐ bachelor (A1)<br>☐ master<br>☐ doctoraat (PhD)

f.	**Welke categorie beantwoordt het best aan uw huidige beroepssituatie?**	☐ Voltijds werk<br>☐ Deeltijds werk<br>☐ Student<br>☐ Momenteel heb ik geen werk<br>☐ Anders: ____________________
g.	**Heeft u uw werksituatie moeten aanpassen naar aanleiding van de ziekte van uw kind?**	☐ Nee<br>☐ Ja, namelijk: ______________________<br>________________________________<br>________________________________
h.	**Wat is uw relatie met het kind?**	☐ Ik ben de biologische moeder<br>☐ Ik ben de biologische vader<br>☐ Ik ben de stiefmoeder/ plusmama<br>☐ Ik ben de stiefvader/ pluspapa<br>☐ Ik heb een relatie met de biologische moeder of vader van het kind<br>☐ ander/niets van bovenstaande opties
i.	**Wat is uw gezinssituatie?** (kies wat best past)	☐ gehuwd of samenwonend<br>☐ gescheiden en alleenwonend<br>☐ weduwe/weduwnaar<br>☐ alleenstaande ouder – ongehuwd<br>☐ nieuw samengesteld gezin<br>☐ ander
j.	**Wat is uw nationaliteit?**	
k.	**Wat is de nationaliteit van uw kind?**	____
l.	**Heeft u nog andere kinderen? Zo ja, hoeveel andere kinderen?**	☐ Nee<br>☐ Ja, ik heb ____ andere kinderen
m.	**Wat zijn de geboortedata van uw andere kinderen?**	____<br>____<br>____
n.	**Bent u gelovig?**	☐ Ja, voeg toe wat uw religieuze overtuiging is (optioneel): __________________________<br>☐ Nee<br>☐ Ik zou deze vraag liever niet beantwoorden

Deel 5: Heeft u nog opmerkingen?

14. Schrijf het hier:

Vragen of opmerkingen? Stuur een mailtje naar
Anne.van.Driessche@vub.be of bel op +32456302963

BOOST: een studie rond vroegtijdige zorgplanning in de kinderoncologie

Vragenlijst voor kinderoncologen (T0)

Waarover gaat het onderzoek?

Deze vragenlijst kadert in een onderzoek van **de Vrije Universiteit Brussel (VUB) en Universiteit Gent (UGent),** gefinancierd door het Fonds Wetenschappelijk Onderzoek (FWO) en het Kinderkankerfonds**.** Het doel van dit project is het evalueren van **voorafgaande of vroegtijdige zorgplanning (VZP) bij adolescenten met kanker, hun ouders en kinderoncologen**. De studie wordt uitgevoerd over een periode van 2 jaar (van 18 januari 2021 – 18 januari 2023) in de vier universitaire ziekenhuizen in Vlaanderen.

Waarom vragen we u deze vragenlijst in te vullen?

Een gezin dat deelneemt aan de studie heeft uw naam opgegeven als de kinderoncoloog met wie ze het meeste contact hebben over de zorg en behandeling van de adolescent. De data collector van het onderzoeksteam heeft u de gegevens van dit gezin per mail bezorgd. Wij zouden u willen verzoeken deze vragenlijst over de adolescent van dit gezin in te vullen, zodat we belangrijke informatie kunnen koppelen aan de vragenlijsten die het gezin heeft ingevuld. U zult later nog 2 uitnodigingen ontvangen om een soortgelijke vragenlijst over deze adolescent in te vullen (verspreid over een periode van 6 maanden).

Als u deze vragenlijst invult, dient u het volgende te weten:

- ✓ Dit project werd opgestart na goedkeuring door de ethische comités van UZ Brussel/VUB, UZ Leuven, UZ Gent en UZ Antwerpen.
- ✓ Uw deelname is geheel **vrijwillig**. **Indien u deze vragenlijst invult,** gaat u ermee akkoord dat de studiegegevens die voor de hier vermelde studie worden verzameld, later zullen worden verwerkt, op voorwaarde dat deze verwerking beperkt blijft tot de context van de hier vermelde studie.
- ✓ De gegevens die in het kader van uw deelname worden verzameld zijn **vertrouwelijk**, in overeenstemming met de Belgische Wetgeving op de bescherming van de persoonlijke levenssfeer.
- ✓ Uw gegevens worden **anoniem** verwerkt. De u persoonlijk toegewezen code, zal enkel door een onafhankelijke data-collector aan uw persoonlijke gegevens gelinkt kunnen worden.

Enkele instructies voor u van start gaat:

1. Het invullen van deze vragenlijst duurt **5 -10 minuten**
2. Alle vragen kunt u beantwoorden door één of meerdere van de vakjes aan te vinken ☑
3. Zou u de vragenlijst **binnen een week** willen invullen?

Vragen?

Contacteer Anne van Driessche (uitvoerend onderzoeker)
tel: +32(0)456302963
e-mail: Anne.van.Driessche@vub.be

Alvast hartelijk bedankt voor uw bereidheid om deze vragenlijst in te vullen!

Vragenlijst over de adolescent	
1. Datum van vandaag:	___/____/___ (dd/mm/jjjj)
2. Wat is de hoofddiagnose van het kind op dit moment?	☐ Leukemie<br>☐ Hersentumor<br>☐ Lymfeklierkanker<br>☐ Neuroblastoom<br>☐ Retinoblastoom<br>☐ Levertumor<br>☐ Niertumor<br>☐ Kiemceltumor<br>☐ Bottumor<br>☐ Tumor van de weke delen<br>☐ Andere:<br>________________________________
3. Is er sprake van een recidief?	☐ Nee<br>☐ Ja, eerste recidief<br>☐ Ja, tweede recidief of meer
4. Is er sprake van metastasering?	☐ Nee<br>☐ Ja (vul in), ____________________________
5. Zijn er comorbiditeiten* bij dit kind? Zo ja, welke?<br><br>*aanwezigheid van een aandoening naast de primaire, centraal staande ziekte	____<br>____<br>____
6. Wanneer is het kind voor de eerste keer gediagnosticeerd met kanker?	Datum: ___/____/___ (dd/mm/jjjj)

7.	**Wat is het type behandeling dat het kind nu krijgt?** (meerdere antwoorden mogelijk)	☐ Chemotherapie<br>☐ Radiotherapie<br>☐ Hormoontherapie<br>☐ Chirurgie<br>☐ Experimentele therapie (Farmacologische en/of non-farmacologische behandeling waarvan het nuttig effect nog dient bewezen te worden in de klinische praktijk. Specificeer:___________________________________)<br>☐ Ik weet het niet<br>☐ Geen behandeling<br>☐ Andere: ______________________________
8.	**Welke lijn behandeling krijgt het kind op dit moment?**	☐ Eerste lijn<br>☐ Tweede lijn<br>☐ Derde lijn of meer
9.	**Wat is het huidige hoofddoel van de behandeling van het kind? Kies wat het meest van toepassing is.**	☐ Patiënt krijgt een behandeling die in opzet curatief is.<br>☐ Patiënt krijgt een behandeling die in opzet levensverlengend is.<br>☐ Patiënt krijgt een behandeling die in opzet symptoombestrijdend is.
10.	**Hoe lang zorgt u al voor dit kind?**	☐ 0 – 6 maanden<br>☐ 6 – 12 maanden<br>☐ 1 – 2 jaar<br>☐ Meer dan 2 jaar
11.	**Zou u de kans op overleving kunnen inschatten voor dit kind op dit moment?** (in percentage)	___%

Hieronder vindt u een aantal vragen over vroegtijdige zorgplanning.

Met vroegtijdige zorgplanning (VZP) bedoelen we het communicatieproces dat kinderen in staat stelt om te bespreken wat voor hen belangrijk is en hen in staat stelt om doelen en voorkeuren te formuleren voor toekomstige zorg en behandeling, deze doelen en voorkeuren te bespreken met familie en zorgverleners en om eventuele voorkeuren vast te leggen en indien nodig te herzien. Voorbeelden van VZP-thema's zijn: beleving van de ziekte, angsten, wie wil je dat er beslissingen maakt en hoe, toekomstverwachtingen, voorkeuren voor zorg en behandeling voor nu en in de toekomst.

12. **Welke thema's heeft u in de afgelopen 30 dagen besproken met dit kind?** (meerdere antwoorden mogelijk)	☐ Zijn/haar angsten of bezorgdheden<br>☐ Zijn/haar verwachtingen voor de toekomst<br>☐ Wat hij/zij belangrijk vindt als het om zijn of haar zorg of behandeling gaat.<br>☐ Keuzes die mogelijks gemaakt zullen moeten worden in de behandeling<br>☐ Wanneer hij/zij het stoppen van de behandeling zou overwegen<br>☐ Wanneer hij/zij het wel of niet starten van een nieuwe behandeling zou overwegen<br>☐ Met wie de behandeling verder besproken moet worden, mocht het kind wilsonbekwaam worden<br>☐ De kans dat hij/zij zou sterven.<br>☐ Andere thema's gerelateerd aan VZP:_________<br>____<br>____<br>☐ Dergelijke thema's zijn nog niet aan bod gekomen **(ga dan naar vraag 14)**<br>☐ Ik heb deze patiënt de afgelopen 30 dagen niet gesproken **(ga dan naar vraag 14)**
13. **Hoe heeft u de verkregen informatie over bovenstaande thema's in vraag 12 van dit kind verwerkt en doorgegeven?** (meerdere antwoorden mogelijk)	☐ Ik heb deze informatie zelf bijgehouden. Ik heb deze niet gedeeld met anderen.<br>☐ Ik heb dit doorgegeven aan één of meerdere leden van het medisch team op een informele manier.<br>☐ Ik heb dit doorgegeven aan het medisch team tijdens een multidisciplinair overleg<br>☐ Ik heb deze informatie toegevoegd aan het medisch dossier<br>☐ Anders, namelijk: ___________________________<br>____

14. Bent u van plan om de komende drie maanden bepaalde thema's te bespreken met dit kind? Zo ja, welke thema's zijn dat? (meerdere antwoorden mogelijk

- ☐ Zijn/haar angsten of bezorgdheden
- ☐ Zijn/haar verwachtingen voor de toekomst
- ☐ Wat hij/zij belangrijk vindt als het om zijn of haar zorg of behandeling gaat.
- ☐ Keuzes die mogelijks gemaakt zullen moeten worden in de behandeling
- ☐ Wanneer hij/zij het stoppen van de behandeling zou overwegen
- ☐ Wanneer hij/zij het wel of niet starten van een nieuwe behandeling zou overwegen
- ☐ Met wie de behandeling verder besproken moet worden, mocht het kind wilsonbekwaam worden
- ☐ De kans dat hij/zij zou sterven.
- ☐ Andere thema's gerelateerd aan VZP:________

  ____

  ____
- ☐ Ik ben niet van plan om dergelijke thema's te bespreken omdat, ________________________

  ____
