# Supplementary material for: Advance care planning for adolescents with cancer and their parents: study protocol of the BOOST pACP multi-centre randomised controlled trial and process evaluation
Source: BMC Pediatr. 2021 Sep 1;21:376. doi: 10.1186/s12887-021-02841-7 (PMC8408307; doi:10.1186/s12887-021-02841-7)
Supplement: Supplementary file 2 — Additional file 2. ‘Process evaluation methods based on UK MRC guidance on process evaluations of complex interventions (Moore et al., 2015)’. This table provides an overview of all process indicators that are evaluated in the process evaluation, including methods and at what timepoints. [file 12887_2021_2841_MOESM2_ESM.docx]

**Additional file 2. *Process evaluation methods based on UK MRC guidance on process evaluations of complex interventions (Moore et al., 2015)***

| **Dimension and subdimension** (definition by Moore et al. 2015) | **Outcomes and indicators (I)**  If no level is indicated the indicator is applicable across levels or no specific level | **Data collection**  each outcome/indicator can be evaluated qualitatively or quantitatively using one or more data-collection methods, see corresponding letters | **Timing**  (T0: baseline, T1: 3 months, T2: 7 months) |
| --- | --- | --- | --- |
| **Implementation *(****the structures, processes and resources through which delivery is achieved, and the quantity and quality of what is delivered)* | | | |
| **1. How delivery is achieved** *(the structures, processes and resources through which delivery is achieved)* | | | |
| **1.A. Structures** (how the intervention activities were implemented) | **(a) description of how the intervention activities were implemented per family**:  I1: location of ACP conversations  I2: order of activities implemented, and how those were implemented  I3: who was present during the ACP conversations  I4: whether and how information is transferred to paediatric oncologist  I5: whether the facilitator had contact with the family in between/after ACP sessions, how (e.g. modes of communication) and about what  I6: whether the facilitator used extra exercises during the ACP, how and why | **(a1)** To measure I1-6, facilitators will complete a structured diary  **(a2)** Semi-structured individual interviews with facilitators | **(a1)** Throughout the data collection period after each facilitated ACP conversation with a patient and/or family  **(a2)** Intermediate (every 6 months) and post data collection period |
| **1B. Resources** | **(b) Time investment facilitators**  **I1:** Time spent on training  **I2:** Time spent by facilitator on preparation of intervention activities  **I3:** Time spent by facilitators on delivery of the intervention activities | **(b)** To measure I1-3, we will use specific questions in the structured diaries to keep track of time spent on preparation and delivery of key ACP activities (cf. conversations) | **(b)** monthly by facilitators, throughout data collection period |
| **2. What is delivered (***the quantity and quality of what is delivered)* | | | |
| **2A. Dose** (*how much intervention/the quantity of what is delivered)* | **(c) Amount of intervention activities delivered per family**  I1: number of ACP activities held per family  I2: number of topics discussed and not discussed during the conversations | **(c1)** To measure I1-I2 we will ask facilitators to complete a structured diary  **(c2)** To measure I2 we will audiotape ACP conversations | **(c1)** Throughout the data collection period after each facilitated ACP conversation with a patient and/or family  **(c2)** Each ACP conversation held is ideally audiotaped (after informed consent of participants) |
| **2B. Reach** *(extent to which a target audience comes into contact with the intervention)* | **(d) Number of eligible families approached by facilitator, and proportion of families included in total**  I1: number of adolescents involved  I2: number of parents involved  I3: average ratio adolescent: parent | **(d)** Trial monitoring | **(d)**Throughout the study |
| **2C. Fidelity** *(consistency of what is implemented with the planned intervention)* | **(e) activities delivered as intended:**  I1: number of activities delivered as intended  I2: order of intervention activities delivered as planned  **(f) content of ACP conversations delivered as intended**  I1: number of ACP conversation sessions in which *(all, half, less than half -* of the topics were discussed | **(e)** To measure I1-2 we will ask facilitators to complete a structured diary  **(f)** To measure I1 we will audiotape ACP conversations; we will use the pre-structured checklist based on conversation manuals to check fidelity (we aim to select 8 families who have participated in all 4 BOOST pACP conversations, from whom we will analyse all 4 conversations. We intend to include dyads from different hospitals and with adolescents from different age categories) | **(e)** Throughout the data collection period after each facilitated ACP conversation with a patient and/or family  **(f)** Each ACP conversation held is ideally audiotaped (after informed consent of participants) |
| **2D. Adaptations** *(alterations made to an intervention in order to achieve better conceptual fit)* | **(g) Type of adaptations made in intervention activities** (as described by manual)  **(h)** **Type of adaptations made in intervention activities per hospital** | **(g1)** Facilitators complete a structured diary  **(g2+h)** Semi-structured individual interviews with facilitators  **(h)** Semi-structured individual interviews with paediatric oncologists (n = 8) | **(g1)** Throughout the data collection period after each facilitated ACP conversation with a patient and/or family  **(g2)** Intermediate (every six months) and after the data collection period.  (**h)** After 12 months and after data collection period |
| **2E. Quality** *(the quality of what is delivered)* | **(i) Perceived quality of the ACP conversations** | **(i)** Semi-structured interviews with adolescent and parent(s) separately (n=10) | **(i)** Post-intervention maximally two months after the last ACP session |
| **Mechanisms of impact** *(the intermediate mechanisms through which intervention activities produce intended (or unintended) effects/how intervention activities, and participants’ interactions with them, trigger change)* | | | |
| **1. Responses and interactions** *(how participants interact with the intervention)* | **(j) Level of satisfaction about the intervention as a whole and the different components**  **(k) Perceived benefit and relevance of the intervention/value placed on the intervention** | **(j1+k1)** Semi-structured interviews with adolescent and parent(s) separately (n=10)  **(j2+k2)** Semi-structured individual interviews with paediatric oncologists(n=8)  **(k3)** Semi-structured interviews with facilitators | **(j1)** Post-intervention maximally two months after the last ACP session  **(j2)** after 12 months and post data collection period  **(k3)** every 6 months and post-intervention |
| **2. Mediators** *(intermediate processes which explain subsequent changes in outcomes)* | **(l) Evaluation of perceived mediators:**  I1: the extent to which BOOST has contributed to more knowledge about ACP  I2: more positive attitude on ACP  I3: better self-efficacy in ACP  I4: a higher intention to communicate on ACP themes  I5: improved communication between …  adolescent, parent(s) and healthcare professionals | **(l1)** Semi-structured interviews with adolescent and parent separately (n= 10)  **(l2)** Semi-structured interviews with facilitators  **(l3)** Semi-structured interviews with paediatric oncologists (n = 8) | **(l1)** post-intervention (in adolescents/parents, maximally two months after the last ACP session)  **(I2)** every 6 months and post-intervention  **(I3**) after 12 months and post-intervention |
| **3. Unanticipated pathways or consequences** | **(m) + (n)** Potential unanticipated consequences of the BOOST pACP program in adolescents, parents (per family) and in oncologists, teams, hospital wards (whether the intervention led to other changes) | **(m)** Semi-structured interviews with adolescent and parent separately (n = 10)  **(n)** Semi-structured interviews with paediatric oncologists(n = 8) | **(m)** post-intervention (in adolescents/parents, maximally two months after the last ACP conversation of the family)  **(n)** after 12 months and post-intervention |
| **Context** *(factors external to the intervention which may influence its implementation, or whether its mechanisms of impact act as intended / how external factors influence the delivery and functioning of interventions)* | | | |
| **1. Contextual moderators** | **(o) + (p)** Contextual barriers that inhibit/facilitators that facilitated pACP implementation and/or effectiveness (eg. skills, attitudes, or organisational norms, culture (human, financial, resources) | (o1+p1) Semi-structured interviews with adolescent and parent separately (n = 10)  (o2+p2) Semi-structured interviews with facilitators  (o3+p3) Semi-structured interviews with paediatric oncologists (n=8) | **(o1 ,p1)** post-intervention (in adolescents/parents)-, maximally two months after the last ACP conversation of the family)  **(o2+p2)** every 6 months and post-intervention  **(o3 + p3**) after 12 months and post-intervention |
| **2. Intention for maintenance** *(extent to which the program is intended to be part of routine organisational practice and policy)* | **(q) + (r)** Intention of adolescents/parents/healthcare professionals to conduct ACP conversations (or other BOOST pACP activities) in the future | (q) Semi-structured interviews with adolescent and parent separately (n = 10)  (r) Semi-structured interviews with paediatric oncologists(n=8) | **(q)** post-intervention (in adolescents/parents)-, maximally two months after the last ACP conversation of the family)  **( r)** after 12 months and post-intervention |
